# Pre-existing resistance in the latent reservoir can compromise VRC01 therapy during chronic HIV-1 infection

**DOI:** 10.1371/journal.pcbi.1008434

**Published:** 2020-11-30

**Authors:** Ananya Saha, Narendra M. Dixit

**Affiliations:** 1 Department of Chemical Engineering, Indian Institute of Science, Bengaluru, India; 2 Centre for Biosystems Science and Engineering, Indian Institute of Science, Bengaluru, India; University of Zurich, SWITZERLAND

## Abstract

Passive immunization with broadly neutralizing antibodies (bNAbs) of HIV-1 appears a promising strategy for eliciting long-term HIV-1 remission. When administered concomitantly with the cessation of antiretroviral therapy (ART) to patients with established viremic control, bNAb therapy is expected to prolong remission. Surprisingly, in clinical trials on chronic HIV-1 patients, the bNAb VRC01 failed to prolong remission substantially. Identifying the cause of this failure is important for improving VRC01-based therapies and unraveling potential vulnerabilities of other bNAbs. In the trials, viremia resurged rapidly in most patients despite suppressive VRC01 concentrations in circulation, suggesting that VRC01 resistance was the likely cause of failure. ART swiftly halts viral replication, precluding the development of resistance during ART. If resistance were to emerge post ART, virological breakthrough would have taken longer than without VRC01 therapy. We hypothesized therefore that VRC01-resistant strains must have been formed before ART initiation, survived ART in latently infected cells, and been activated during VRC01 therapy, causing treatment failure. Current assays preclude testing this hypothesis experimentally. We developed a mathematical model based on the hypothesis and challenged it with available clinical data. The model integrated within-host HIV-1 evolution, stochastic latency reactivation, and viral dynamics with multiple-dose VRC01 pharmacokinetics. The model predicted that single but not higher VRC01-resistant mutants would pre-exist in the latent reservoir. We constructed a virtual patient population that parsimoniously recapitulated inter-patient variations. Model predictions with this population quantitatively captured data of VRC01 failure from clinical trials, presenting strong evidence supporting the hypothesis. We attributed VRC01 failure to single-mutant VRC01-resistant proviruses in the latent reservoir triggering viral recrudescence, particularly when VRC01 was at trough levels. Pre-existing resistant proviruses in the latent reservoir may similarly compromise other bNAbs. Our study provides a framework for designing bNAb-based therapeutic protocols that would avert such failure and maximize HIV-1 remission.

## Author summary

Antiretroviral therapy (ART) can control but not eradicate HIV-1. Stopping ART leads to rapid viral resurgence and progressive disease. ART is therefore administered lifelong. Tremendous efforts are ongoing to devise strategies that will enable stopping ART and yet prevent viral resurgence. One such strategy involves the administration of broadly neutralizing antibodies (bNAbs) of HIV-1 at the time of stopping ART. This strategy is expected to delay if not prevent viral resurgence. Surprisingly, treatment with VRC01, a potent bNAb, resulted in hardly any improvement in viral remission. In this study, we elucidate the cause of this failure. We hypothesized that VRC01-resistant strains may pre-exist in latently infected cells, which are unaffected by ART. They can thus outlast ART and get reactivated, triggering VRC01 failure. We built a detailed mathematical model based on this hypothesis and showed that it quantitatively captured observations of VRC01 failure in clinical trials on chronic HIV-1 patients. Our study thus identifies a potential vulnerability of bNAbs, namely, bNAb-resistant strains pre-existing in latently infected cells. Our model offers a framework for predicting bNAb-based treatment protocols that would preclude failure due to pre-existing resistance and maximally prolong remission.

## Introduction

Antiretroviral therapy (ART) for HIV-1 infection rapidly suppresses viremia to undetectable levels and curtails disease progression but is unable to eradicate the virus [1]. Discontinuation of ART, even long after viremic control is established, typically leads to rapid viral rebound, often within days to weeks of discontinuation, and to progressive disease [2]. The rebound is caused by a reservoir of latently infected cells [3] that is formed soon after infection [4]. The reservoir is sustained by cell proliferation [5,6], which can continue even when ART has stopped new infections, allowing the reservoir to exist long-term [7]. The reservoir can get reactivated stochastically and reignite infection once ART is stopped [8,9]. ART must therefore be administered lifelong. In a remarkable breakthrough, the VISCONTI study showed that when ART is administered early in infection, some individuals can maintain viremic control for many years after the cessation of ART [10]. This study has raised hopes of a functional cure, or long-term remission, of HIV-1, where the viremic control once established by ART can be maintained without lifelong treatment [11]. The success of ART in inducing post-treatment control, however, is small: only ~5–15% of the patients treated achieve lasting post-treatment control [12]. Enormous efforts are underway to improve this success rate [11].

One strategy that holds promise is to administer broadly neutralizing antibodies (bNAbs) of HIV-1 for a short period post-ART, i.e., during an analytical treatment interruption (ATI) [13,14]. bNAbs target diverse viral genomic variants [15,16], and are expected to suppress viremia arising from the reactivation of latently infected cells [17,18]. Simultaneously, they may engage the host immune system [19], potentiating it to maintain the viremic control long-term [20,21]. Two recent clinical trials tested this strategy using the bNAb VRC01 [17]. VRC01 targets the CD4 binding site on the HIV-1 envelope with high breadth and potency [15,22]. When it was administered to chronically infected patients who had achieved undetectable viremia with ART, the duration of viremic control was observed to increase only marginally, by a median of ~2–4 weeks, beyond historical controls [17]. In the historical control group, which was not treated post-ART, viral rebound occurred in ~2.6 weeks on average after ART interruption [23]. Why VRC01 was ineffective in maintaining remission longer is unclear. Unravelling the causes of this ineffectiveness is important to optimizing VRC01 usage, which is also in large clinical trials for preventing the transmission of infection [24,25], and to expose potential vulnerabilities of other bNAbs.

VRC01 exhibits potent antiviral activity *in vivo* [26], including at its trough levels in patients [27]. Yet, in the trials above [17], viral rebound was observed in most patients when VRC01 levels in circulation were significantly higher than its *in vitro* suppressive concentration (or *IC*_50_), implicating the role of resistance. Mutations that confer resistance to bNAbs are well documented [28–31]. Indeed, VRC01-resistant strains were detected in the breakthrough viral populations in the trials above [17]. The rapid virological breakthrough during treatment, especially given the absence of circulating virions at the time of ART cessation and the potent activity of VRC01 against the wild-type, suggests that VRC01 resistance might have existed before VRC01 therapy. Here, we therefore hypothesized that pre-existing VRC01-resistant proviruses formed before ART and contained in the latent reservoir could underlie the failure of VRC01 therapy. Minority viral variants as well as latently infected cells are difficult to detect using current assays [32,33]. Testing our hypothesis experimentally would require detecting minority variants ‘within’ latently infected cells, highlighting the challenge involved. Current assays can rarely detect variants below a frequency of ~1% [34], implying that, given the prevalent estimates of the latent reservoir size of 10^5^–10^8^ cells [4,35], variants present in as many as 10^3^ latently infected cells may go undetected and be responsible for therapy failure. Indeed, in many individuals who failed rapidly in the trials above [17], viral genome sequencing could not detect VRC01 resistance pre-treatment [17,36]. As an alternative approach, therefore, we resorted to mathematical modeling.

A number of mathematical models have been developed in recent years to describe latent cell infection and dynamics and its role in the outcomes of treatments [37–48]. Models have also been constructed, independently, for describing viral evolution and drug resistance, especially in the context of ART [49–53]. Describing the failure of VRC01 therapy required integrating these two independent formalisms, of viral evolution and latent reservoir reactivation, a task not accomplished so far because of the complexity involved. HIV-1 evolution involves mutation, recombination, fitness selection, and random genetic drift, which together define the timing and speed of the development of drug resistance during ART [51–55]. Latent cell reactivation is an intrinsically stochastic process [8,45,46], following which the virions released must establish lasting infection, which is not guaranteed [37,39,42], especially in the presence of bNAbs. Here, we developed a framework that integrates these processes by recognizing that the dynamics of viral evolution and latent cell reactivation could be decoupled in the context of post-ART bNAb therapy. Viral evolution primarily occurs pre-ART, where viral and productively infected cell populations are large and the contribution from latently infected cells to the dynamics can be ignored. Latent cell reactivation leading to treatment failure occurs during bNAb therapy, when active viral replication is small and so viral evolution can be ignored. These processes are linked by VRC01 resistant strains, which are predominantly formed before therapy and are harbored in latently infected cells and could get reactivated during therapy. Developing a model with this strategy, we were able, for the first time, to capture data from human clinical trials involving bNAb-based interventions quantitatively, offering an explanation of the inadequate effectiveness of VRC01 in maintaining remission, and providing a framework for rational treatment optimization.

## Results

### Mathematical model

**Fig 1:**
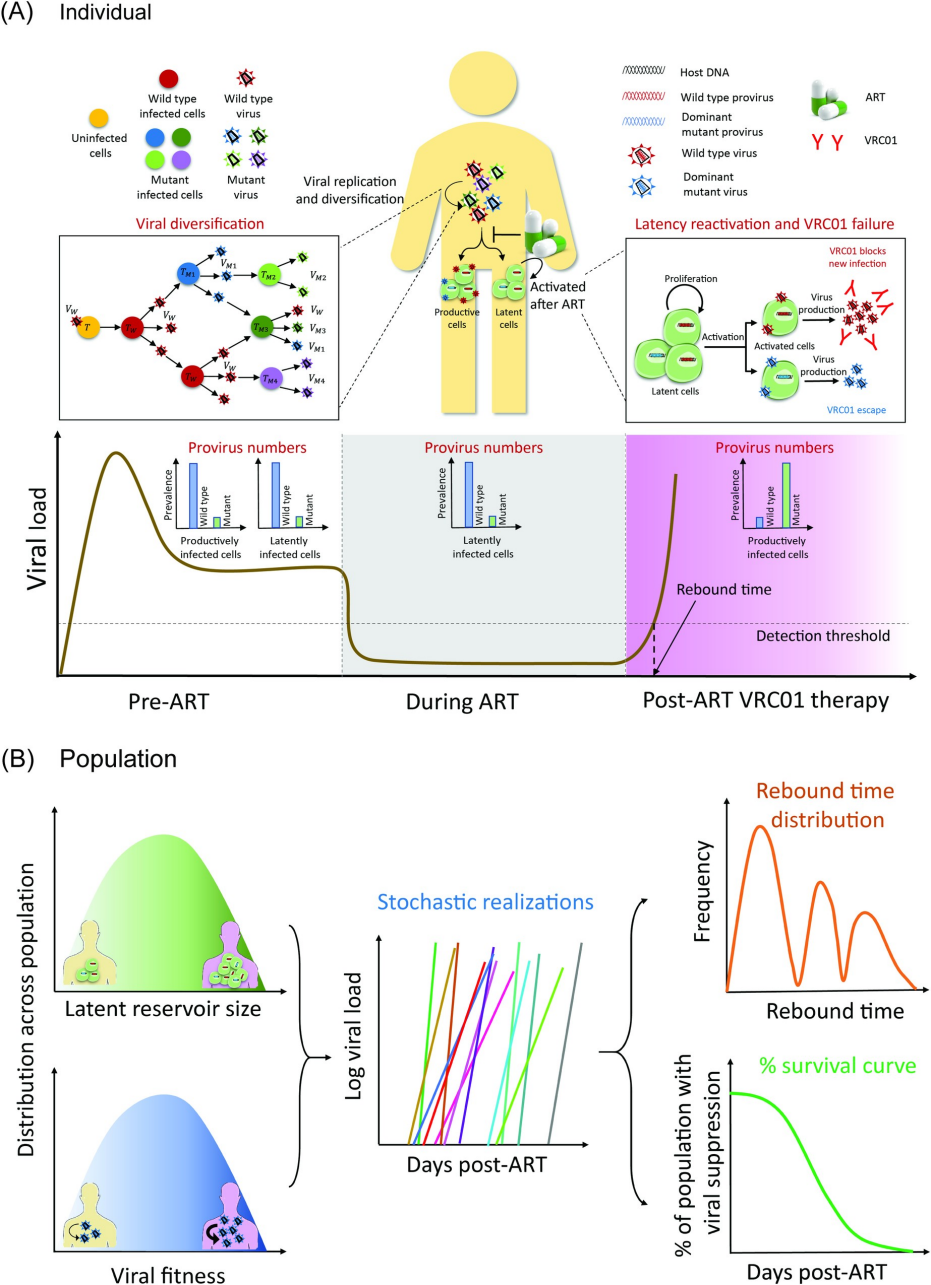
Overview of the model. **(A)** Dynamics at the individual patient level. We used a model of within-host HIV-1 evolution to estimate the pre-ART frequencies of wild-type and VRC01-resistant mutants (left), letting them be identical in productively and latently infected cells. ART eliminates the former but not the latter cells (middle). We then used a stochastic model of latent cell reactivation and viral growth to estimate the time of virological failure following VRC01 therapy (right) for a given size of the latent reservoir and the fitness of the VRC01 resistant strain. **(B)** Dynamics at the patient population level and the outcomes of clinical trials. We created a virtual patient population by sampling the latent cell pool size and mutant viral fitness during VRC01 therapy from defined distributions (left). For each individual, we performed stochastic simulations as in (A) and estimated the time to virological failure (middle), from which we obtained the distribution of breakthrough times and Kaplan-Meier survival plots (right), which we compared with clinical data.

We considered the scenario where chronically infected individuals with viremic control established with long-term ART are administered VRC01 during an ATI, as in recent clinical trials [17]. We developed a model to predict the ensuing remission times based on the hypothesis that viral strains resistant to VRC01 harbored in latently infected cells were responsible for virological breakthrough (Fig 1). We provide an overview of the model here; details are in Methods.

We first considered a single infected individual (Fig 1A). We estimated the diversity of the viral population in the individual at ART initiation using a detailed model of viral dynamics and evolution that considered target cells, free virions, and productively infected cells, and included mutation, cellular superinfection, recombination, and fitness selection based on the relative fitness of dominant VRC01 resistance mutations. From the diversity, we obtained the frequencies of productively infected cells containing different mutant proviruses resistant to VRC01. We let the latent reservoir harbor proviruses with the same frequencies, as has been done previously [55]. We recognized that of the latter cells, those harboring the most frequent mutant provirus were the most likely to re-establish infection. We employed a stochastic model to estimate the waiting time for the reactivation of such latent cells and tracked the ensuing dynamics during VRC01 therapy until the infection grew to detectable levels, at which point the therapy was deemed to have failed. During the simulations, we let the efficacy of VRC01 vary continuously with time based on its pharmacokinetic profile, which we estimated from independent fits to data. The parameter values employed for estimating the proviral frequencies and latent cell reactivation are listed in Tables 1 and 2, respectively.

**Table 1:** Parameter values used in the model (Eqs 1–10) for estimating mutant frequencies.

Parameter	Description	Value	Source
*λ*	Production rate of uninfected cells	10^5^ cells/mL/day	[52]
*d*_*T*_	Per capita death rate of uninfected cells	0.1/day	
*k*_0_	Infection rate constant for uninfected cells	2.4×10^−8^ mL/day	
*k*_1_	Cellular superinfection rate constant	0.7*k*_0_	
*δ*	Per capita death rate of productively infected cells	1/day	
*p*	Viral production rate	4,792 virions/cell/day	
*c*	Virion clearance rate	23/day	
*μ*	Mutation rate	3×10^−5^ substitutions/site/replication	
*ρ*	Recombination rate	8.3×10^−4^ crossovers/site/replication	
*β*_*i*_	Relative infectivity	Listed in S1 Table	[28]
*ξ*_*i*_	Replicative fitness	Listed in S1 Table	S1 Fig

**Table 2:** Parameters values used in the model of viral rebound (Eqs 11–20). Parameters not listed are in Table 1.

Parameter	Description	Value	Source
*L*_0_	Initial latent reservoir size	*Log*_10_(*L*_0_)~*N*(6.3,0.43) cells (lognormal distribution)	S3 Fig
*ρ*_*l*_	Per capita proliferation rate of latently infected cells*	0.0042/day	[39,41]
*d*_*l*_	Per capita death rate of latently infected cells*	0.004/day	[39]
*a*_*c*_	Per capita activation rate of latently infected cells*	7.8×10^−4^/day	[39,41]
*f*	Fraction of new infections that lead to latency	10^−6^	[39]
*k*	Infectivity^$^	1.6×10^−12^/day	[52]
*p*	Viral production rate	*p*~*N*(2100,200) virions/cell/day (normal distribution)	[41,44]
*U*	Uninfected cell population^$^	1.5×10^10^ cells	[52]
*ε*_*ART*_	ART efficacy	0.99	Assumed
*IC*_50,*m*_	50% inhibitory concentration for the N279K mutant^&^	800 *μg/mL*	S4 Fig
*A*_1_	Portion of VRC01 dose associated with first phase decay^#^	451 (320, 580) *μg/mL*	Best-fit (Fig 4A inset)
*A*_2_	Portion of VRC01 dose associated with second phase decay^#^	1253 (910, 1596) *μg/mL*	
*η*_1_	First phase decay rate^#^	0.093 (0.078, 0.109) /day	
*η*_2_	Second phase decay rate^#^	1.374 (0.752, 1.995) /day	

* *ρ*_*l*_, *a*_*c*_, and *d*_*l*_ together yield a half-life of ~40 months for the latent pool, consistent with experiments [7].

^$^Corresponding to 15 L of body fluid volume.

^&^Estimates indicate a value much larger than 50 *μg*/*mL* [25,28], but a precise value is lacking.

^#^ 95% confidence limits are in brackets.

Next, we constructed a large virtual patient population based on inter-patient variations in the size of the latent reservoir and the fitness of mutant viral strains, to reflect variations in host and viral factors, respectively, that could influence the outcomes of therapy (Fig 1B). We applied our model above to each virtual patient and estimated the time of therapy failure. From these simulations, we estimated the distribution of remission times in the population and constructed Kaplan-Meier survival plots. We used data from one clinical trial to estimate the parameters defining the inter-patient variations in the virtual patient population and used them without adjustable parameters to predict the outcomes of another clinical trial, validating our model and the parameter estimates.

### Frequencies of VRC01-resistant mutants pre-existing in the latent reservoir

**Fig 2:**
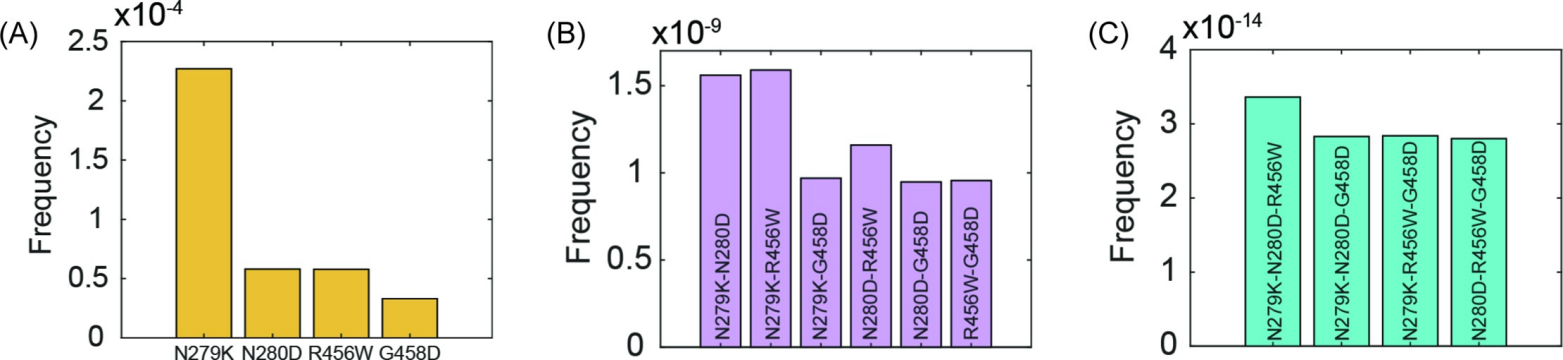
Pre-existing frequencies of mutants. Frequencies of **(A)** single mutants, **(B)** double mutants, and **(C)** triple mutants resistant to VRC01 estimated by our model (Methods). The frequencies, including of the wild-type and the quadruple mutant, not shown here, are listed in S1 Table.

To estimate the frequencies of VRC01 resistant proviruses that may exist in the latent reservoir and cause VRC01 failure, we considered viral evolution before the initiation of ART in a chronically infected individual. During ART, viral replication is quickly halted [5,6], leaving little scope for further viral diversification. We considered four mutations in the HIV-1 envelope region reported to be highly resistant to VRC01: N279K, N280D, R456W and G458D [28]. Resistance could come from strains carrying these mutations singly, in pairs, in triplets, or all together. We considered all these strains in addition to the wild-type, or VRC01 sensitive, strain in our model. The frequencies of the strains would depend on their relative fitness, which, following previous studies [56–58], is defined in our model by two components: infectivity and replicative ability. The relative infectivity of each of the strains involved has been estimated in independent experiments [28], which we employed (S1 Table). The replicative abilities can be estimated using competitive growth assays [59], which have been reported for some of the strains [28]. From the assays, the two fittest single mutant strains, N279K and N280D, appeared to have replicative fitness not significantly different from the wild-type [28]. We analysed data available from the assays for the other two single mutants, R456W and G458D, and the quadruple mutant [28] using a previously developed formalism [59] and estimated their replicative fitness (S1 Fig). For all the double (except N279K-R456W, which had replicative fitness similar to the wild type [28]) and triple mutants, as an approximation, we set the replicative fitness to values predicted assuming zero epistasis. (This assumption is not critical to our findings, which, as we show below, depend primarily on the single mutant frequencies.) The fitness values are in S1 Table. The fitness values were consistent with recent deep mutational scanning analyses, which suggest that most mutations in the HIV-1 envelope are under purifying selection [60]. With these fitness values and all other parameters representative of chronic HIV-1 infection (Table 1), we estimated the frequencies of productively infected cells harbouring the different mutant proviral genomes (Fig 2).

Our model predicted that the fittest mutant strain, N279K, would exist at a frequency of approximately 2.3×10^−4^, the highest among the different mutants (Fig 2A). Other single mutants were at frequencies ~5-fold lower. The double mutants had frequencies of ~10^−9^ (Fig 2B), triple mutants of ~10^−14^ (Fig 2C), and the quadruple mutant of ~10^−19^ (S1 Table). These frequencies are similar but not identical to those expected from the mutation-selection balance, where a strain with *n* mutations would exist at a frequency of ~(*μ*/*a*)^*n*^, with *a* the constant per mutation fitness penalty and *μ* the mutation rate [49]. For N279K, for instance, where *a*~0.14, considering both relative replicative fitness and relative infectivity (S1 Table), and with *μ*~3×10^−5^ [52], the mutation-selection balance would yield a frequency of 2.14×10^−4^, slightly lower than that predicted by our model.

Following previous models, where a constant fraction of infection events is assumed to lead to latency [39,42,47], the frequencies of the mutants are expected to be similar in productively and latently infected cells. The latent reservoir is not affected by ART directly, and even in the absence of active viral replication, the latent reservoir would decrease in size extremely slowly, taking years [7]. Thus, one could safely assume that the latent reservoir at the initiation of ART would exist nearly intact post-ART and at the start of VRC01 therapy, as also suggested by recent observations [61]. We do recognize that the latent reservoir can harbour genomes from early in the infection [61], so that the estimate diversity in the plasma would be an upper bound on the diversity in the latent reservoir. Current technologies have been unable to estimate the diversity in the latent reservoir comprehensively [36]. We therefore employ the upper bound as a conservative estimate. Given the latent reservoir size, *L*_0_, of ~10^5^–10^8^ cells in chronically infected individuals [35,39], the expected number of cells infected with the N279K mutant proviruses would be ~23–23000. The corresponding numbers would be ~10–5000 for the other single mutants (Fig 2). For the double and triple mutants, however, the numbers would be <0.1 and <10^−6^, respectively. On average, thus, our calculations predicted that most latently infected cells would carry wild-type, or VRC01-sensitive proviruses. A small number, ~10–10^4^ cells, would carry single mutants resistant to VRC01. Cells carrying higher mutants were unlikely to exist. The single mutants, too, may not be detectable in most cases, given current assay limits (see Introduction).

With this description of the frequencies of VRC01 resistant proviruses in the latent cell reservoir, we examined the dynamics of VRC01 failure due to latent cell reactivation.

### *de novo* generation of mutants during VRC01 therapy

From the above estimates, the lower end of the spectrum of latent cell numbers carrying single mutant proviruses, ~10 cells, is small enough that it is possible that in some individuals, due to stochastic variations in the mutant frequencies and/or infected cell numbers, the latent reservoir contains no resistant proviruses. In such a scenario, *de novo* mutation after the reactivation of latent cells carrying VRC01 sensitive proviruses would have to give rise to resistance during VRC01 therapy. Even in the presence of latent cells carrying single mutant proviruses, the large majority of cells carrying VRC01 sensitive strains may result in *de novo* mutations following reactivation of latent cells carrying VRC01 sensitive proviruses being the predominant mechanism of VRC01 failure. We developed a model to test this possibility (S1 Text) and found that the estimated virological breakthrough times were far larger (over ~100 days) than those observed clinically (~20 days) (S2 Fig). Although reactivation of such cells was more frequent, given their larger numbers, than the cells carrying mutant proviruses, such reactivation did not lead to lasting infection in our predictions because VRC01 successfully neutralized the viruses produced. Mutations, being intrinsically rare, did not lead to rapid enough *de novo* development of resistance. The VRC01 failure seen clinically was thus unlikely to be due to the reactivation of latently infected cells carrying VRC01 sensitive proviruses. The more likely mechanism therefore was the reactivation of latently infected cells carrying single mutant proviruses resistant to VRC01. We estimated remission times based on the latter mechanism next.

### Growth of pre-existing mutants during VRC01 therapy

**Fig 3:**
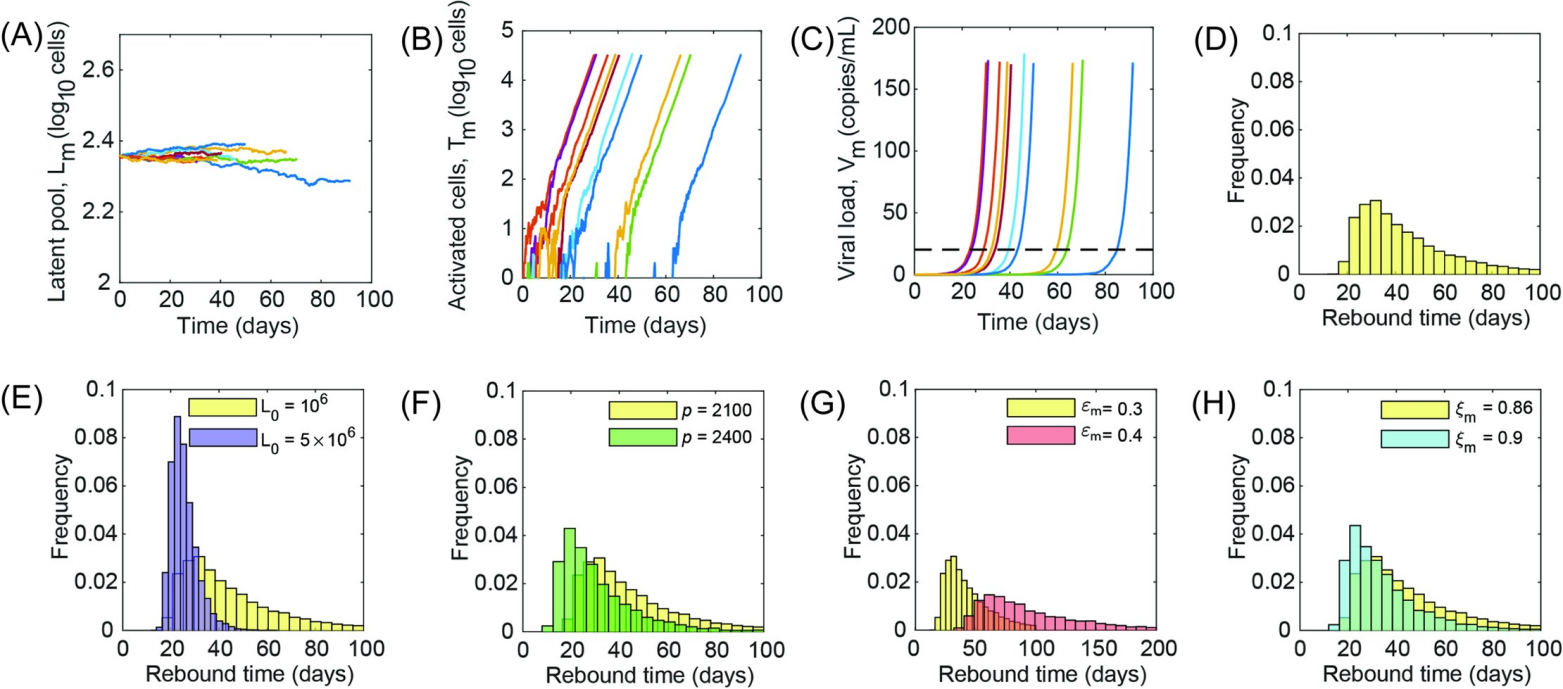
Dynamics of VRC01 failure due to pre-existing resistance. Representative trajectories of **(A)** the latent cell pool harboring resistant proviruses, **(B)** activated cells, and **(C)** VRC01-resistant viral load, obtained by our stochastic simulations (Methods). The different colors represent individual trajectories. Black dashed line shows the detection limit, crossing which marks clinical rebound. **(D)** The distribution of rebound times obtained from 5000 realizations. Here, the initial population of latently infected cells carrying the resistant mutants was set to 2.3×10^−4^×*L*_0_, where *L*_0_ = 10^6^ cells, and the VRC01 efficacy against the mutant to *ε*_*m*_ = 0.3. Other parameters used are in Tables 1 and 2. Variation of the distribution is shown with **(E)** initial latent pool, *L*_0_ (cells), **(F)** viral production rate (virions/cell/day), **(G)** VRC01 efficacy, and **(H)** mutant fitness.

We focussed on latently infected cells carrying proviruses with the N279K mutation, which, with their 5-fold higher prevalence than other single mutants, were the most likely to be reactivated. Stochastic simulations (Methods) with a constant VRC01 efficacy against the mutant, indicated that the latent cell pool did not vary significantly over the durations considered (Fig 3A), consistent with experiments [36]. Reactivation leading to the growth of productively infected cells carrying proviruses with the N279K mutation occurred over a duration of a few days to weeks (Fig 3B). Detectable viremia, however, took longer given that the viral levels had to rise to 20 copies/ml (Fig 3C). Defining the time for viremia to become detectable as the time of the failure of VRC01 therapy, or the breakthrough time, the simulations yielded a distribution of breakthrough times ranging from 20–100 d when *L*_0_ was 10^6^ cells (Fig 3D), consistent with observations [17], suggesting that virological breakthrough was likely to be due to the reactivation of cells infected latently with single mutant VRC01-resistant strains.

The breakthrough time depended on the time for latent cell reactivation as well as for the subsequent establishment of successful infection. Thus, higher *L*_0_, which decreased reactivation times, led to earlier breakthrough (Fig 3E). Increasing the viral production rate (Fig 3F), lowering drug efficacy ((Fig 3G) or increasing viral fitness (Fig 3H), which improved the chances of establishment of successful infection after reactivation, all led to more rapid virological breakthrough. These factors are likely to vary across individuals. We examined next how their influence would manifest in clinical trials and whether our simulations could capture the VRC01 failure seen in the trials.

### Multimodal distribution of breakthrough times in a virtual patient population

**Fig 4:**
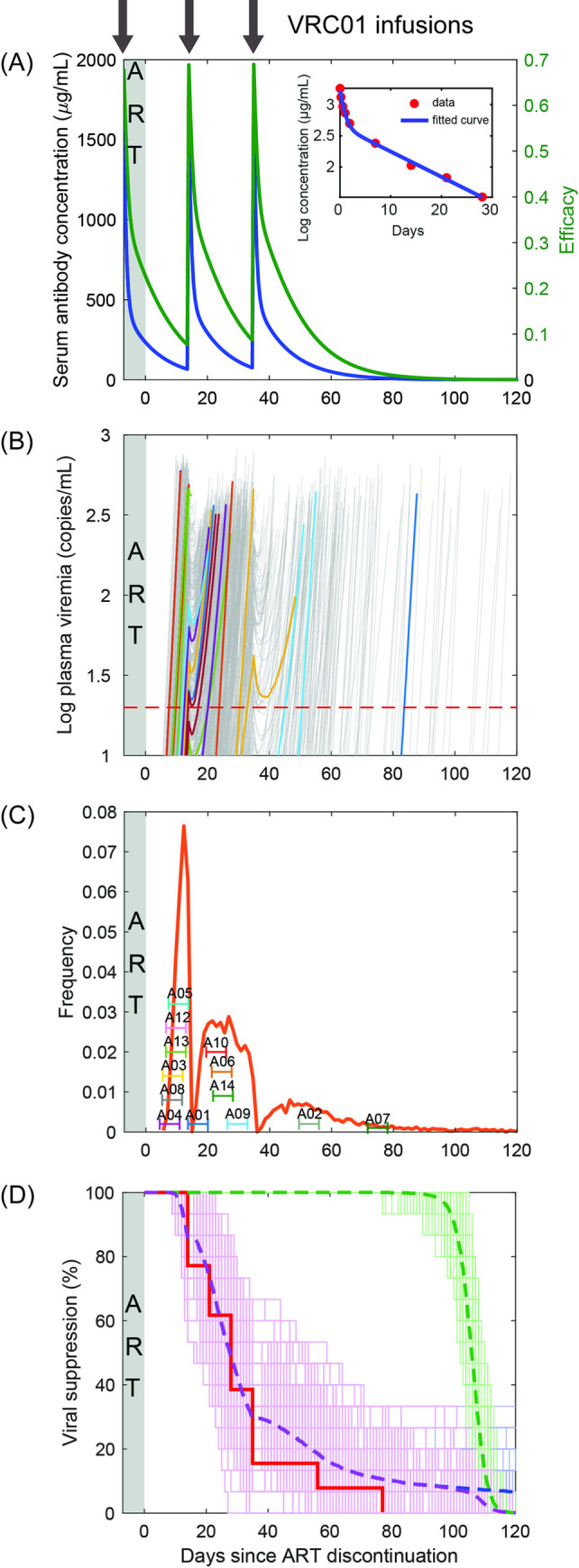
Recapitulating VRC01 failure in the A5340 trial. **(A)** Fits of our model of single-dose plasma VRC01 pharmacokinetics (line) to data [26] (symbols) (*Inset*). Best-fit parameter estimates are in Table 2. Corresponding multiple dose concentration profiles (blue) and the VRC01 efficacy against a mutant strain with *IC*_50_ = 800 *μg*/*mL* (green) are shown. Black arrows indicate VRC01 infusions. **(B)** Stochastic realizations of the dynamics in a virtual population of 10000 patients, manifesting as changes in plasma viremia. Each grey line represents a virtual patient. Some randomly chosen trajectories are colored to aid visualization of the dynamics. Note that ART was continued after the first infusion of VRC01 for 1 week. The detection limit of 20 copies/mL is marked as a red dashed line, crossing which marks clinical viral rebound. **(C)** The corresponding distribution of rebound times (orange). Rebound times of the participants in the A5340 trial with a 1-week uncertainty period, representing the gap between successive viral load measurements, are also marked. **(D)** Kaplan-Meier plot for the A5340 trial based on the percentage of patients with viremia ≥200 copies/mL. Each light purple line is a survival curve generated by randomly choosing 20 patients from the virtual population above. The dashed purple line is the mean of all these survival curves. Each light green line is an analogous survival curve if the failure were to occur by the recrudescence of latently infected cells infected by wild-type (VRC01 sensitive) proviruses. (*IC*_50_ for the wild-type was 1 *μg*/*mL* [25].) The green dashed line represents the average of the latter survival curves. The blue dashed line marks the average survival curve due to resistant strains alone. It is indistinguishable from the purple line until ~100 days, indicating that the predominant mode of failure is via resistance. The data from the trial [17] is shown as a red solid line.

We focused here on the A5340 trial [17] where VRC01 therapy was initiated a week before the end of ART on patients with well-controlled viremia. 3 doses of VRC01 were administered, with a gap of 3 weeks between successive doses. Virological breakthrough was detected when the viral load crossed 20 copies/ml. To mimic the trials, it was necessary not only to account for inter-patient variations but also to consider time-varying efficacies of drugs within an individual, which can significantly affect the development of drug resistance [51,62]. We therefore first considered VRC01 pharmacokinetics and fit a model of biphasic decay to data of the VRC01 plasma concentration profile following a single intravenous dose [26] (Fig 4A inset). Using the resulting best-fit parameters, we predicted the multiple dose pharmacokinetics for the dosing protocol above and estimated the time-varying efficacy, *ε*_*m*_ (Fig 4A), which we employed in our stochastic simulations.

Next, we created a virtual patient population to mimic inter-patient variations expected in clinical trials. A number of host factors, including HLA types, are known to affect the ability of individuals to control viremia [63]. Similarly, viral factors have also been argued to determine the level of viremia in chronic infection [64]. A convolution of host and viral factors is expected to determine clinical outcomes. The specific factors involved and how they vary across individuals, however, is not fully established [46,63]. Here, we employed a parsimonious approach where we let two parameters, one reflective of variations in host factors and the other viral factors, define the inter-patient variations. We thus constructed a virtual patient population with different initial latent pool sizes, *L*_0_, subsuming variations in all host-factors, and viral production rates, *p*, subsuming variations in all viral factors. For each individual, we sampled *L*_0_ and *p* independently from pre-defined distributions (Methods) and ran a stochastic simulation to describe the ensuing dynamics.

The simulated dynamics showed virological breakthrough times varying from a few days to a few months post cessation of ART across individuals (Fig 4B). Following breakthrough, the viral load rose sharply. It was suppressed partially following the administration of a VRC01 dose, but then rose again once the VRC01 level fell. The patterns were similar to the viral load resurgence patterns seen in patients [17]. From the breakthrough data, we estimated the distribution of virological breakthrough times in this virtual patient population (Fig 4C). We found that the distribution was multimodal. Until a week or so following the cessation of ART, no breakthrough was expected based on the distribution because of high VRC01 levels in circulation. As the VRC01 concentration declined, breakthrough began. The distribution of breakthrough times peaked when the VRC01 concentration was at its trough level, just before the administration of the second VRC01 dose. Following dosing, the distribution dropped steeply, and rose again as the VRC01 concentration waned. It then attained a peak that was smaller than the first peak and began to fall subsequently. The smaller size of the peak and the subsequent fall was due to a convolution of the effect of VRC01 and the natural distribution of breakthrough times in the absence of treatment. Post ART cessation, it has been shown that the distribution of mean rebound times is unimodal and declines following its peak [41]. In other words, after the peak, the population of individuals that suffers breakthrough decreases with the breakthrough time. In our simulations, this decline is what explains the smaller second peak compared to the first and the drop in the distribution after the second peak although the VRC01 levels were low. Of course, with the third dose, the distribution dropped sharply again due to the rise in the VRC01 level and then rose as the VRC01 level waned. The distribution attained a third peak that was even smaller than the second peak and ended in a long tail representing the small fraction of individuals who experienced longer remission times than studied in our simulations.

Based on the distribution, we constructed a Kaplan-Meier survival plot, which at each time point marked the percentage of the virtual patient population that was still under remission, defined as viremia <200 copies/mL [17]. The plot, as expected, indicated no failure for a short period, ~1–2 weeks, after ART, then dropped sharply, reaching 50% failures in about 4 weeks, and displayed a long tail with a small percentage, ~10%, maintaining remission for longer than the duration of our simulations (100 days) (Fig 4D). We examined next whether these predictions could recapitulate clinical observations.

### Model calibration to recapitulate the A5340 trial

Comparing our simulations with data required knowledge of the distributions of *L*_0_ and *p* in patients. We chose *p* to mimic viral growth rates after rebound. The growth rate can vary from 0.4–1.5/day [39,42]. We therefore chose the mean value of *p* to yield a growth rate of 1/day. Further, we let *p* follow a normal distribution with a standard deviation that ensured positive growth rate across two standard deviations from the mean. The resulting distribution of *p* is mentioned in Table 2. Although estimates of the variations in *L*_0_ exist [35,39], in our simulations, they had to accurately mimic the variations in the pool carrying resistant proviruses, which are not known. We therefore adopted the following approach. We decided to employ data from the A5340 trial to estimate parameters characterizing the distributions of *L*_0_ and then validate them using an independent trial, the NIH trial. Both the trials involved small sample sizes, ~10–15 patients [17]. Non-linear mixed effects modeling, designed particularly to estimate parameter distributions using clinical data from small sample sizes, works with deterministic but not stochastic models [65,66]. Consequently, we adopted a heuristic approach to estimate the distributions of *L*_0_. We recognized that the product *a*_*c*_*L*_0_, with *a*_*c*_ the latency reactivation rate, determines the waiting time for viral recrudescence; the larger the product, the earlier would be the reactivation of the latent reservoir. We fixed *a*_*c*_ based on previous estimates [39,47]. (Note that previous studies report a wide range for *a*_*c*_ [37,39,42].) Through small test simulations, we identified approximate values of *L*_0_ that mimicked the mean waiting times seen in patients in the A5340 trial. We then performed more detailed simulations by varying the distribution of *L*_0_ around the approximate parameters and identified those distributions that best described the Kaplan-Meier survival data from the A5340 trial (S3 Fig). The resulting parameters are also in Table 2. Similarly, we explored the implications of variations in the *IC*_50_ of VRC01 against the mutant and found that values ≥800 *μg/mL* captured the data well (S4 Fig). (We recognize that *IC*_50_ values of rebound virus in individual patients were lower [17]. These could be due to differences between *in vitro* and *in vivo* estimates [67], possible evolution post-breakthrough in response to autologous antibodies [36], and/or inter-patient variations (S5 Fig).)

Simulations with the resulting distributions recapitulated data from the A5340 trial. We found that 11 of the 12 patients who experienced treatment failure despite high concentrations of VRC01 in circulation [17] had breakthrough times close to the peaks in the multimodal distribution of breakthrough times we predicted (Fig 4C). The number of patients with breakthrough times associated with the different peaks was also proportional to the area under the peaks. Note that the area under a peak is a measure of the probability, and hence the frequency, of failure corresponding to the times associated with the peak. 6 patients failed during the times associated with the first peak, 4 with the second peak, and one with the third peak. The areas under the peaks from our simulations yielded failure percentages of ~35%, ~44%, and ~21%, respectively. The first two peaks, thus, appeared to have comparable failure percentages, whereas the third was substantially smaller. Given the small sample size in the clinical trial, the distribution of patients into the three peaks appeared to be consistent with the estimated failure percentages. One patient (A01) appeared to fail at the trough in the distribution after the first peak, and this could be due to stochastic effects or variations not captured in our virtual population.

Kaplan-Meier plots based on breakthrough times, *i*.*e*., times for the viral load to reach 200 copies/ml, from the virtual patient population also closely recapitulated the clinical data (Fig 4D). Here, to account for the small sample size in the trials, we chose many samples of 20 individuals each, selected randomly from our virtual patient population, and constructed Kaplan-Meier curves for each sample. In addition to failure due to VRC01 resistant strains, for each virtual patient we also estimated the breakthrough time due to the wild-type, or VRC01 sensitive, strains (S1 Text), and set the breakthrough time of the individual to be the smaller of the two. The clinical data fell within the ranges defined by these curves (purple lines in Fig 4D). Further, the mean of these curves was in close agreement with the data. Accordingly, 50% of the treated population exhibited a breakthrough time of >4 weeks from the end of ART (or 5 weeks from the start of VRC01 therapy), consistent with the clinical data. Importantly, the simulations predicted that the failure was predominantly due to VRC01 resistant strains. Ignoring failure due to the wild-type strains made little difference to the survival plot (compare blue and purple lines in Fig 4D). The failure due to the wild-type strains became important only after VRC01 levels had declined significantly (green lines in Fig 4D). The simulations tended to marginally over-predict the clinical data for long remission times; whereas all the patients had shown rebound by 80 d, the simulations predicted that ~10% patients would retain control still and take much longer (up to ~120 *d*) to experience failure. We attributed this to the presence of individuals with strong immune responses and/or small latent cell populations, including post-treatment controllers [63], who may not be seen in the small population of 13 individuals in the A5340 trial. Notwithstanding these deviations, our simulations closely recapitulated the data from the A5340 trial.

### Model validation with the NIH trial

**Fig 5:**
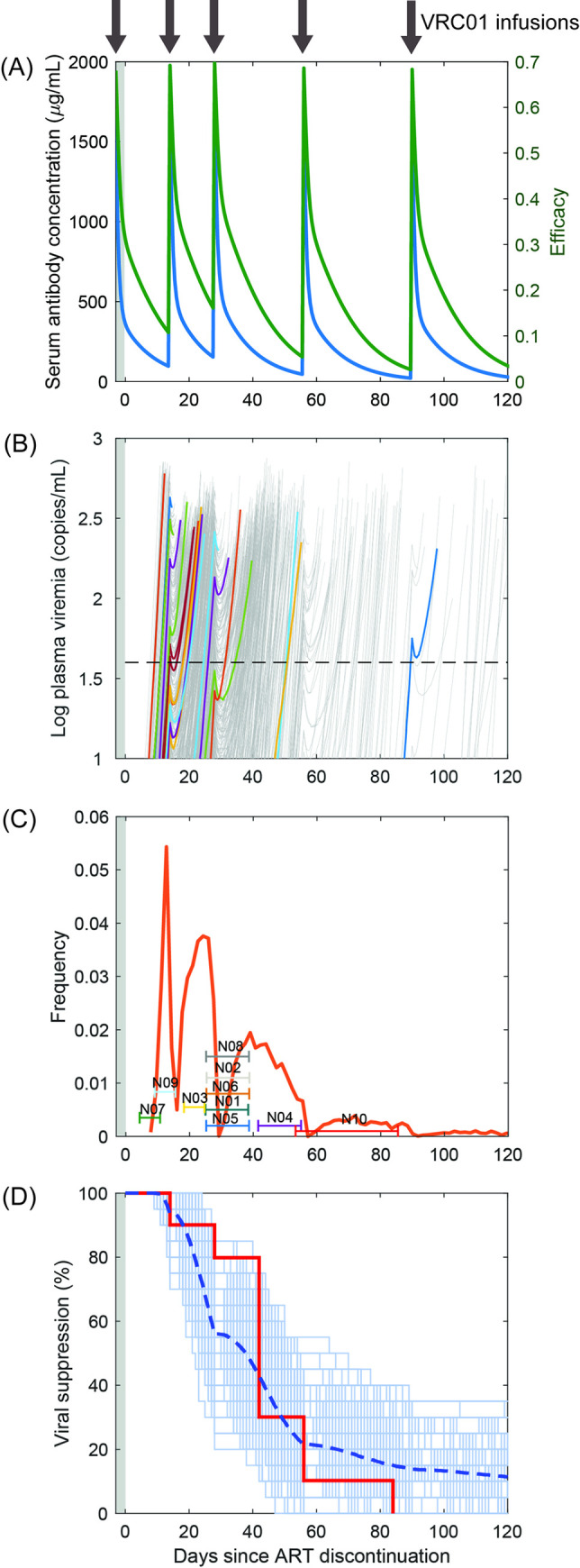
Recapitulating VRC01 failure in the NIH trial. **(A)** Multiple dose concentration profiles (blue) and the VRC01 efficacy against a mutant strain with *IC*_50_ = 800 *μg/mL* (green). Black arrows indicate VRC01 infusions. **(B)** Stochastic realizations of the dynamics in a virtual population of 10000 patients, manifesting as changes in plasma viremia. Each grey line represents a virtual patient. Some randomly chosen trajectories are colored to aid visualization of the dynamics. ART was continued after the first infusion of VRC01 for 3 days (grey shaded region). The detection limit of 40 copies /mL is marked as a black dashed line, crossing which marks clinical viral rebound. **(C)** The corresponding distribution of rebound times (orange). Rebound times of the participants in the NIH trial are marked along with their uncertainties based on measurement frequencies. **(D)** Kaplan-Meier plot for the NIH trial. Each light blue line is a survival curve generated by randomly choosing 20 patients from the virtual population above. The dashed blue line is the mean of all these survival curves. The data from the trial is shown as a red solid line.

To test and validate our model and the parameter estimates, we applied our simulations to describe a second, independent clinical trial, the NIH trial [17], where 10 individuals were subjected to VRC01 therapy during an ATI. Treatment commenced 3 days before ART cessation. Subsequent doses were administered on weeks 2 and 4 and then every month until 6 months. To describe the resulting breakthrough data, we created a virtual population exactly as above and subjected it to therapy following the clinical protocol. VRC01 pharmacokinetics was predicted based on the corresponding dosing times (Fig 5A). All the parameters were kept the same as those as in our simulations of the A5340 trial.

We found that viral rebound trajectories were similar to those in the A5340 trial, with sharp rises in viral load following breakthrough and wide inter-patient variations (Fig 5B). The distribution of breakthrough times again followed a multimodal distribution with the peak widths broadening progressively with each dose and culminating in a long tail (Fig 5C). Based on the areas under the peaks, the percentages of failure at times corresponding to the four peaks were~16%, ~39%, ~34%, and ~9%, respectively. Indeed, in agreement, 7 of the 10 patients failed at times corresponding to the second and third peaks. 2 patients were associated with the first peak and one with the last. More precise timing of failure of the patients was not possible given the much larger intervals between successive viral load measurements in the NIH trial compared to the A5340 trial. Nonetheless, the agreement between our simulations and the observations of the distribution of failure times between peaks, given the small sample size, was remarkable. We also computed Kaplan-Meier survival plots, using samples of 20 virtual patients from our virtual population, and considering failure due to VRC01 resistant strains. (Failure due to the wild-type was unlikely, as observed above.) The Kaplan-Meier plots captured the clinical data quantitatively (Fig 5D), indicating that our simulations accurately mimicked the response of patients to VRC01 therapy in the NIH trial.

The difference between the A5340 trial and the NIH trial was in the dosing protocol alone. Indeed, once our model was calibrated with data from the A5340 trail, it captured the data from the NIH trial by simply changing the dosing protocol accordingly, and required no other adjustments, thereby providing a strong test and validation of our model. The model may be applied to predict the outcomes of other possible dosing protocols, which could involve changing dosages or half-lives (S6 Fig), providing a framework for rational therapy optimization.

## Discussion

Passive immunization with HIV-1 bNAbs holds promise as a strategy to achieve long-term remission of HIV-1 infection [20,68]. Following its success in macaques [20,21], enormous efforts are underway to translate it to humans [69]. Trials with VRC01 administered to chronically infected patients following the cessation of successful ART saw rapid virological failure despite the presence of suppressive concentrations of VRC01 in circulation [17], signaling a potential vulnerability of such bNAb therapies. Here, using mathematical modeling and analysis of clinical data, we elucidated the likely cause of this rapid VRC01 failure. Model predictions attribute this failure to the reactivation during therapy of cells latently infected with VRC01 resistant proviruses before ART initiation. To arrive at this inference, we constructed a mathematical model that integrated within-host viral evolution, latency reactivation, and viral dynamics with VRC01 pharmacokinetics, and applied it to simulate the outcomes of therapy in a virtual patient population. Our simulations recapitulated data from clinical trials [17], giving us confidence in the inference. Accounting for pre-existing resistant strains in the latent reservoir would be important to the success of bNAb-based therapies.

That mutation-driven resistance can be an important cause of bNAb failure has been recognized earlier [28–31]. Indeed, efforts are ongoing to identify bNAbs, including in the VRC01 class, that are less vulnerable to such resistance [70]. For bNAb therapies that target maintenance of HIV remission post ART cessation during chronic infection, our study prescribes the requisite genetic barrier to resistance, *i*.*e*., the number of mutations HIV-1 must accumulate to develop significant resistance to the therapy. Using a detailed description of within-host HIV-1 evolution, our study predicts that with the estimated latently infected reservoir size of 10^5^–10^8^ cells in a chronically infected individual [35,39], proviral strains carrying single but not more resistance mutations would pre-exist in the latent reservoir. Consequently, a genetic barrier of 2 would be necessary to prevent pre-existing resistance from compromising therapy. A bNAb with a genetic barrier of one, like VRC01, would be predestined to fail, as was observed in clinical trials [17]. When used in combination with another bNAb, however, the overall barrier would cross the threshold of 2, diminishing the chances of such failure. Resistance would then have to develop by *de novo* mutation during therapy, which according to our model would be unlikely as long as adequate bNAb concentrations exist in circulation and restrict the replication of the wild-type virus. Further, by explicitly incorporating bNAb pharmacokinetics, our model provides a framework with which optimal dosing protocols could be identified that would ensure adequate bNAb concentrations throughout.

Early ART initiation has been argued to restrict the viral reservoir size and improve the chances of post-treatment control [10,47]. Our study predicts an additional advantage of early ART initiation: The chances of failure due to resistance are reduced. This reduction happens in multiple ways. First, most HIV-1 infections involve a single founder virus, which gradually evolves into the diverse quasispecies seen in chronic infection [54,71,72]. Thus, the frequency of mutants at the time of ART initiation is expected to be smaller, the earlier the initiation [54]. Second, given the limited viral diversity early in infection, the latent reservoir is likely to have a much higher representation of bNAb-sensitive strains than estimated using our model of chronic infection. Finally, if the reservoir size is restricted, due to early ART initiation, far fewer mutant proviruses, or none at all, may exist in the latent reservoir. Together, thus, early initiation of ART is expected to reduce the chances of bNAb failure. Indeed, in trials where ART was initiated early, in the acute phase of infection, no genotypic resistance to VRC01 was detected from sequence analysis of viral strains post failure [73]. Similarly, a combination of two bNAbs, including one in the VRC01 class, administered early in infection saw no resistance to therapy in SHIV-infected macaques [20]. Surprisingly, however, the breakthrough times for VRC01 therapy following ART in acute infection were found to be similar to those seen in the trials in chronic infection we studied [17,73]. Additional mechanisms that are not predominant in the chronic phase thus appear to cause VRC01 therapy failure in individuals treated with ART early. One possibility could be that CD8 T cell exhaustion is weaker and/or more reversible in the acute phase than in the chronic phase because of the much longer duration of antigen exposure in the latter scenario [74,75]. The greater associated immune activation levels in the acute phase could imply more rapid reactivation of latently infected cells, which could offset the advantage from the lower frequencies of mutants. To test this possibility, models that incorporate CD8 T cell exhaustion [39,76–79] would have to be integrated with our model of stochastic latency reactivation, a promising avenue for future study.

An important question in HIV cure research is how early should ART be initiated to maximize post-treatment control [63]. While starting early would restrict the reservoir size and improve the chances of post-treatment control, starting it too early would not allow enough time for the development of an immune response, compromising post-treatment control. If bNAb therapy were to be used post-ART to improve the chances of post-treatment control, the development of resistance to bNAbs would have to be factored in along with the latter trade-off between reservoir size and immune response strength to arrive at the optimal timing of ART initiation. Our study provides a framework that could be used to test whether pre-existing resistance in the latent reservoir would compromise therapies with other bNAbs, especially those belonging to the VRC01-class [80–82], which are under trial for both preventive [24,83,84] and therapeutic [20] vaccination and are known to fail via diverse mutation-driven resistance pathways [29].

A modeling study several years ago compared the likelihood of the failure of therapy due to pre-existing resistance versus *de novo* generation of mutants in the context of ART and found that ART was more likely to fail due to pre-existing resistance [50]. A more recent modeling study examined the likelihood of the failure of different antiretroviral drug combinations and explained how patient adherence influences such failure [55]. Our findings are consistent with these studies and show that bNAbs too are vulnerable to pre-existing resistance, but the resistance now is restricted to the latent reservoir. Because the reservoir is small compared to the pool of infected cells pre-ART, the required genetic barrier for bNAb therapies is estimated to be lower than for ART. Thus, a combination of 2 bNAbs is predicted to overcome resistance, whereas first-line ART necessarily contains 3 drugs.

Previous studies have used either fully deterministic or fully stochastic frameworks to describe treatment failure [50,51,55], making their predictions approximate or computationally expensive, respectively. Here, we devised a strategy that retained both accuracy and computational tractability. We used a deterministic framework to estimate the frequencies of mutant proviral genomes in the latent reservoir pre-treatment and then a stochastic framework to estimate latency reactivation and treatment failure times. The deterministic framework was shown previously to agree well with stochastic population genetics-based simulations [52], giving us confidence in the strategy. In the stochastic simulations, we considered only latently infected cells containing the dominant resistant provirus, which accurately described the development of drug resistance without requiring expensive computations. Indeed, we were able to capture clinical trials of VRC01 failure with this hybrid framework, reiterating its applicability. Such hybrid deterministic-stochastic frameworks have been successfully applied in other settings, such as in predicting the pre-existing frequency of hepatitis C virus strains resistant to drugs [85], but not, to our knowledge, with HIV-1 infection.

The sample sizes involved in the clinical trials we studied were small, ~10–15 patients each [17]. Nonlinear mixed-effects models have been used in recent studies to infer the effects of interventions by analyzing data from such trials [86–88]. For instance, bNAb therapy was argued to improve immune responses 8-fold over controls and not to synergize with the TLR7-agonist [86]. The strength of such an approach lies in its robust parameter estimation and the ability to infer effects at the population level using data from small sample sizes. The approach, however, does not work when the underlying model is stochastic, which is the case in our study. Also, when the effects are explicitly parameterized, as is often done [86,88], their quantitative estimates are restricted to the specific conditions studied. Thus, for instance, how a change in the dosage or dosing protocol would alter the effect becomes difficult to predict. Our approach, being fully mechanistic, is not similarly limited. Indeed, with the parameters that captured the A5340 trial, by simply changing the dosing protocol, our model captured data from the NIH trial without any adjustable parameters. Our model could therefore be used potentially to comparatively evaluate alternative treatment protocols and suggest optimal ones.

We recognize that in the trials we considered [17], the dominant mutant seen post treatment failure was not the same across individuals. The mutations, however, were all typically concentrated in the same genomic regions [17], suggesting that structural or conformational modifications that could drive VRC01 resistance could be produced by many mutations in the same genomic region. While we have focused on the most frequent mutant, as it is the most likely to have caused resistance, and as has been done in earlier studies [55], stochastic variations could result in latent cells carrying other mutants being reactivated and reestablishing infection. The mutants selected could also differ based on inter-host variations and the genetic backgrounds of the infecting strains. Our simulations must thus be viewed as reflecting therapy failure arising from the dominant mutant which could differ across hosts and which in our study is accounted for by the inter-host variation in viral fitness.

bNAbs are known to engage the immune system via multiple mechanisms [20,87,89–91]. The result could be heightened activation, which for HIV-1 infection, could mean greater susceptibility of target CD4 T cells. At the same time, it could imply greater reactivation rates of latently infected cells. Indeed, the latency reactivation rates employed in our study were higher than those estimated for historical controls, the latter based on viral recrudescence post ART and in the absence of further intervention [39]. When bNAb therapy is administered post ART, cells latently infected with wild-type strains, which would be in a vast majority, would get frequently reactivated and produce virions but without causing sustained infection. The virions produced, being bNAb sensitive, would get neutralized and cleared by the bNAbs. Previous studies have argued that in the process bNAbs can stimulate CD8 T cells [20,87], improving the overall immune activation status, possibly explaining the higher latency reactivation rate we required to describe VRC01 failure than previously used to describe historical controls [38]. Our parameters would thus tend to underpredict the remission times in the historical controls. Previously, too, differences in the viral growth rates before and after ART have been assumed in order to capture viral recrudescence accurately and have been attributed to different strengths of the immune responses in the respective periods [39]. Mechanisms that could explain the differences in these latency reactivation rates are yet to be identified. A unified framework that describes remission both with and without bNAb therapy awaits future studies that would quantify how bNAbs influence immune responses.

Non-nucleoside reverse transcriptase inhibitors (NNRTIs), administered as part of ART, have been found to delay breakthrough post ART [23]. Patients administered NNRTIs were excluded from the A5340 trial and were switched to an integrase-inhibitor based regimen 2 weeks before VRC01 therapy in the NIH trial to eliminate the confounding effects of NNRTIs. We therefore did not consider the effect of NNRTIs in our study. A number of host [63] and viral [64] factors are thought to be involved in determining disease progression and treatment outcomes. Our model subsumed inter-patient variations in these factors into variations in two factors, the latent pool size and the viral production rate. Virtual patient populations that we created based on variations in these minimal factors captured clinical data from clinical trials, justifying the approximation, and suggesting that a small subset of factors may be adequate to capture outcomes of such bNAb therapies. Future studies may consider the variations in other factors explicitly to ascertain the validity of the approximation, especially for other kinds of bNAb-based interventions.

In summary, our study presents an explanation of the failure of VRC01 therapy to sustain HIV remission post ART, captures clinical data quantitatively, highlights the importance of accounting for pre-existing resistance in designing effective bNAb-based therapies, and facilitates rational optimization of such therapies.

## Methods

We considered the scenario where a chronically infected individual maintains viral suppression with ART and is then subjected to VRC01 therapy concomitantly with cessation of ART. Because active viral replication is halted by successful ART, virological breakthrough during VRC01 therapy must arise from the reactivation of latently infected cells. We developed a model to estimate the timing of the failure of VRC01 therapy via the growth of resistant viral mutants pre-existing in the latent reservoir (Fig 1). Our approach was to estimate the population of latently infected cells carrying the resistant strains and then to follow their reactivation leading to successful infection. We then considered a virtual patient population subjected to the same therapy and applied our model to recapitulate data from clinical trials.

### Mathematical model of virological breakthrough in a single infected individual

#### Within-host HIV-1 evolution and the frequencies of pre-existing resistant strains

To estimate the population of cells latently infected with VRC01-resistant proviral genomes, we reasoned that the frequencies would be the same as in productively infected cells before treatment initiation because the probability that a particular cell becomes latently infected is not known to depend on the nucleotides at the VRC01 resistance loci. We estimated the frequencies of VRC01-resistant strains in productively infected cells using an approach developed previously to quantify the pre-existence of resistance to antiretroviral drugs [52]. The approach combines virus dynamics with evolution, incorporating mutation, cellular superinfection, recombination, and fitness selection. We present details below.

*Virus dynamics*. We considered *n* = 4 positions in the *env* region where mutations with high level resistance to VRC01 have been identified, namely N279K, N280D, R456W and G458D [28]. The time evolution of the populations of cells and virions containing the different single, double, triple, and quadruple mutants were described using the following equations:

$$\frac{dU}{dt}=\lambda -d_{T}U-k_{0}U\sum_{h=j}^{S}\sum_{j=0}^{S}\beta_{jh}V_{jh}$$ (1)

$$\frac{dT_{i}}{dt}=k_{0}U\sum_{h=j}^{S}\sum_{j=0}^{S}Q_{i}(jh)\beta_{jh}V_{jh}-k_{1}T_{i}\sum_{h=j}^{S}\sum_{j=0}^{S}\beta_{jh}V_{jh}-\delta T_{i}$$ (2)

$$\frac{dT_{ii}}{dt}=k_{1}T_{i}\sum_{m=k}^{S}\sum_{k=0}^{S}Q_{i}(km)\beta_{km}V_{km}-\delta T_{ii}$$ (3)

$$\frac{dT_{ij}}{dt}=k_{1}T_{i}\sum_{m=k}^{S}\sum_{k=0}^{S}Q_{j}(km)\beta_{km}V_{km}+k_{1}T_{j}\sum_{m=k}^{S}\sum_{k=0}^{S}Q_{i}(km)\beta_{km}V_{km}-\delta T_{ij}$$ (4)

$$\frac{dV_{ii}}{dt}=p\xi_{i}(T_{i}+T_{ii})+\frac{p}{2}\{\sum_{h=0}^{i-1}\frac{\xi_{i}^{2}}{\xi_{h}+\xi_{i}}T_{hi}+\sum_{h=i+1}^{S}\frac{\xi_{i}^{2}}{\xi_{i}+\xi_{h}}T_{ih}\}-cV_{ii}$$ (5)

$$\frac{dV_{ij}}{dt}=p\frac{\xi_{i}\xi_{j}}{\xi_{j}+\xi_{i}}T_{ij}-cV_{ij}$$ (6)

Here, uninfected target CD4+ T cells, *U*, are produced from the thymus at the rate *λ* and are lost at the per capita death rate *d*_*T*_. They are infected by virions *V*_*jh*_ containing genomes *j* and *h* with the second order rate constant *k*_0_*β*_*jh*_, where *k*_0_ is the infectivity of wild-type virions and *β*_*jh*_ is the relative infectivity of virions *V*_*jh*_. We assumed that *β*_*jh*_ = (*β*_*j*_+*β*_*h*_)/2, where *β*_*j*_ is the infectivity of genome *j* relative to the wild-type. The indices *j* and *h* denoting viral genomes range from 0 to *S* = 2^*n*^−1. Thus, *S* = 15 here, with the different genomes including the wild-type (or sensitive strain), the 4 single mutants, 6 double mutants, 4 triple mutants, and the quadruple mutant, amounting to a total of 16 types. We denoted the genomes serially, starting with ‘0’ for the wild-type and ending with *S* for the quadruple mutant. Each viral particle contains two genomes, not necessarily identical. The number of distinct viral particle types, *V*_*jh*_, where *j*∈{0,1,..,*S*} and *h*∈{*j*,*j*+1,..,*S*}, is thus (*S*+1)(*S*+2)/2, which here would be 136. (The range of values of *h* is to ensure that virions *V*_12_ and *V*_21_, for instance, which are identical, are not counted separately.) Summing over all these viral types yields the total rate of loss of *U* due to infections in Eq 1.

Following infection with a virion *V*_*jh*_, reverse transcription, which includes mutation and recombination, yields genome *i* with a probability *Q*_*i*_(*jh*), where *i*∈{0,1,..,*S*}. On average, thus, a fraction *Q*_*i*_(*jh*) of the infections with virions *V*_*jh*_ yield productively infected cells carrying single proviruses *i*, which we denote *T*_*i*_. Summing over all *V*_*jh*_ yields the total rate at which cells *T*_*i*_ are produced. These cells die at the per capita rate *δ*. They can also be infected again, but with a lower infectivity *k*_1_ because infected cells downregulate their CD4 receptors, rendering further infections difficult [92,93]. The net effect of these processes defines the dynamics of cells *T*_*i*_ in Eq 2.

Doubly infected cells *T*_*ii*_ are produced when cells *T*_*i*_ are infected with virions *V*_*jh*_, following which reverse transcription again yields the provirus *i*. When a different provirus *j*(≠*i*) is produced, the result is the doubly infected cell *T*_*ij*_ carrying distinct proviruses *i* and *j*. Of course, cells *T*_*ij*_ can also be produced by the infections of cells *T*_*j*_ with another virion yielding the provirus *i*. Doubly infected cells too die with the rate constant *δ*. These processes are contained in Eqs 3 and 4. We neglected cells infected more than twice, following experiments that suggest a rare occurrence of such cellular superinfection [94].

Cells *T*_*i*_ and *T*_*ii*_ produce virions *V*_*ii*_ at the per capita rate *pξ*_*i*_, where *p* is the production rate of wild-type virions and *ξ*_*i*_ the production rate of virions *V*_*ii*_ relative the wild-type. *ξ*_*i*_ is thus the relative replicative fitness of genome *i*. Cells *T*_*hi*_ (*h*≠*i*) can also produce virions *V*_*ii*_. We assumed that viral RNA of the types *h* and *i* are present in cells *T*_*hi*_ in proportion to *ξ*_*h*_ and *ξ*_*i*_, respectively, and that they are randomly assorted into pairs and packaged into progeny virions. Thus, cells *T*_*hi*_ produce virions *V*_*ii*_ at the rate $p\xi_{i}^{2}/2(\xi_{h}+\xi_{i})$, *V*_*hh*_ at the rate $p\xi_{h}^{2}/2(\xi_{h}+\xi_{i})$, and *V*_*ih*_ at the rate *pξ*_*h*_*ξ*_*i*_/(*ξ*_*h*_+*ξ*_*i*_). Virions are cleared at the rate *c*. These processes determine the dynamics of viral populations in Eqs 5 and 6.

*Mutation and recombination*. We next describe the formalism to compute the probability *Q*_*i*_(*jh*). Following previous studies [51,52,95,96], we let *R*_*k*_(*jh*) be the probability that genome *k* is produced by the recombination of genomes *j* and *h*, and *P*_*ik*_ the probability that genome *k* mutates to genome *i*. Thus, *P*_*ik*_*R*_*k*_(*jh*) is the probability that genome *i* is produced from genomes *j* and *h* via the intermediate *k*. We recognize next that if genomes *j* and *h* differ in *d* positions, then recombination could produce a total of 2^*d*^ different genomes, depending on whether at each of the *d* positions, the nucleotide chosen is either from genome *j* or genome *h*. Summing over these different intermediates *k* yields the total probability of producing genome *i* from genomes *j* and *h* during reverse transcription:

$$Q_{i}(jh)=\sum_{k=0}^{2^{d}-1}P_{ik}R_{k}(jh)$$ (7)

To compute *R*_*k*_(*jh*), we consider the desired path of the enzyme reverse transcriptase on the two genomes so that the enzyme is on the appropriate genome, *j* or *h*, at each of the *d* distinctive sites, so that the genome *k* is produced. We let the separation between the *x*^*th*^ and *x*+1^*st*^ distinctive sites be *l*_*x*_. If the enzyme has to be on the same genome (*j* or *h*) at both these sites, then it must perform an even number of crossovers in the length *l*_*x*_. Else, it must perform an odd number of crossovers. If the enzyme has a probability *ρ* of crossover per site, then the probabilities of even and odd crossovers over a length *l* are *P*_*even*_(*l*) = (1+(1−2*ρ*)^*l*^)/2 and *P*_*odd*_(*l*) = (1−(1−2*ρ*)^*l*^)/2, assuming that the crossover at any position is independent of the others and all crossovers happen with the same probability [95]. If we write *P*_*des*_(*x*+1|*x*) as the probability that the enzyme arrives on to the desired genome at the *x*+1^*st*^ distinctive site given that it was on the desired genome at the *x*^*th*^ site, then depending on whether the associated crossovers must be even or odd, we write *P*_*des*_(*x*+1|*x*) = *P*_*even*_(*l*_*x*_) or *P*_*des*_(*x*+1|*x*) = *P*_*odd*_(*l*_*x*_). Note that *P*_*des*_(1) = 1/2 because the enzyme could be on either genome at the start of the reverse transcription process. Thus, multiplying the probabilities over all the distinctive sites yields *R*_*k*_(*jh*):

$$R_{k}(jh)=\frac{1}{2}\prod_{x=1}^{d-1}P_{des}(x+1|x)$$ (8)

Next, we estimated the probability of producing genome *i* from genome *k*. If the two genomes differ at *u* sites, then genome *i* is produced from genome *k* by mutating genome *k* at the distinctive sites and nowhere else. Thus,

$$P_{ik}=\mu^{u}{\left(1-\mu \right)}^{n-u}$$ (9)

where *μ* is the per site mutation probability and *n* is the number of sites of interest.

*Frequencies.* Eqs 1–9 yield a model of viral dynamics that predicts the growth of the populations of different mutants. We solved the equations for their steady states and obtained the corresponding frequencies of all proviruses, *ϕ*_*i*_, contained in productively infected cells:

$$\phi_{i}=\frac{T_{i}+2T_{ii}+\sum_{j=0}^{i-1}T_{ji}+\sum_{j=i+1}^{S}T_{ij}}{\sum_{i=0}^{S}T_{i}+2\sum_{j=i}^{S}\sum_{i=0}^{S}T_{ij}}\approx \frac{T_{i}}{\sum_{i=0}^{S}T_{i}}$$ (10)

The approximation in Eq 10 is justified by the small doubly infected cell population compared to the singly infected cell population. The parameter values employed are in Table 1. *ϕ*_*i*_ yield the frequencies of various VRC01-resistant mutants before the start of treatment. Previous studies have shown that this deterministic formalism yields mutant frequencies in agreement with stochastic population genetics-based simulations of HIV evolution [52]. The frequencies are expected to hold also for the proviruses in latently infected cells. Further, we expect ART not to influence the latter frequencies; standard first-line ART drugs target HIV reverse transcriptase and protease, whereas VRC01 targets the HIV envelope. We assumed therefore that following ART, the frequencies of VRC01-resistant strains contained in the latent reservoir are given by Eq 10.

We focused next on the reactivation of latently infected cells carrying the resistant genomes.

#### Latency reactivation and viral rebound

We developed a stochastic framework to describe the reactivation of latently infected cells carrying mutant proviruses resistant to VRC01. Because reactivation is most likely of the cells carrying the fittest mutant, which are the most prevalent, we considered cells *L*_*m*_ carrying the fittest mutant strain above. Previous studies on ART resistance too have considered the dominant mutant alone [55]. The reactivation of these cells and the ensuing growth of mutant virions is then described by the following events:

$$L_{m}\overset{\rho_{l}}{\to }L_{m}+L_{m}$$ (11)

$$L_{m}\overset{d_{l}}{\to }\phi$$ (12)

$$L_{m}\overset{a_{c}}{\to }T_{m}$$ (13)

$$T_{m}\overset{X_{m}(1-f)(1-\varepsilon_{m})(1-\mu )}{\to }T_{m}+T_{m}$$ (14)

$$T_{m}\overset{X_{m}f(1-\varepsilon_{m})(1-\mu )}{\to }L_{m}+T_{m}$$ (15)

$$T_{m}\overset{\delta }{\to }\phi$$ (16)

Here, cells *L*_*m*_ proliferate at the per capita rate *ρ*_*l*_ (Eq 11), die at the per capita rate *d*_*l*_ (Eq 12), and get activated to productively infected cells, *T*_*m*_, at the per capita rate *a*_*c*_ (Eq 13). Cells *T*_*m*_ carry the mutant provirus and produce resistant virions, *V*_*mm*_, at the per capita rate *pξ*_*m*_, which in turn infect uninfected cells, *U*, with the rate constant *kβ*_*mm*_. Here, *ξ*_*m*_ and *β*_*mm*_ are the relative replicative ability and infectivity, respectively, of the strain *m*. Free virions are cleared at the per capita rate *c*. Viral production and clearance are typically rapid [97] compared to changes in cell populations, so that following an approximation widely used (e.g., see [50]), *V*_*mm*_ can be assumed to be in pseudo-state with *T*_*m*_. Thus, *V*_*nm*_≈*pξ*_*m*_*T*_*m*_/*c*. The rate, *kβ*_*mm*_*VU*, of the infection of *U* thus becomes *kβ*_*mm*_*pξ*_*m*_*T*_*m*_*U*/*c*. We recognized that *U* is not altered significantly due to new infections, especially following ART when the viremia is small. We let a fraction *f* of the new infections lead to latency and the remaining to productive infection. Further, we let a VRC01-resistant strain mutate back to the wild-type with a probability *μ*, the point mutation rate of HIV-1. The resulting description would capture scenarios where a single mutation is adequate to develop resistance, as is the case with VRC01 [28]. Thus, the rate *kβ*_*mm*_*pξ*_*m*_*T*_*m*_*U*(1−*f*)(1−*μ*)/*c* becomes the rate of the growth of *T*_*m*_ in the absence of therapy. If VRC01 were to block infections with efficacy *ε*_*m*_, the rate would become *X*_*m*_*T*_*m*_(1−*f*)(1−*μ*)(1−*ε*_*m*_) with *X*_*m*_ = *kβ*_*mm*_*pξ*_*m*_*U*/*c*. Thus, we let *T*_*m*_ (effectively) double with the per capita rate *X*_*m*_(1−*f*)(1−*μ*)(1−*ε*_*m*_) (Eq 14), and yield new latently infected cells at the per capita rate *X*_*m*_*f*(1−*μ*)(1−*ε*_*m*_) (Eq 15). Cells *T*_*m*_ die with the rate constant *δ*. These events together describe the growth of resistance to VRC01 and therapy failure.

We solved the model equations using the Gillespie algorithm [98] with parameter values representative of HIV-1 infection in the presence of VRC01 therapy. The model was implemented using a program written in MATLAB (S2 Text). The parameter estimates are listed in Tables 1 and 2. The initial population of *L*_*m*_ was set by the frequencies of mutants estimated. With each parameter setting, we performed 5000 realizations to examine the dynamics of treatment failure.

### Comparison with clinical data

We applied our model to predict the outcomes of trials with VRC01 [17]. For this, we explicitly considered VRC01 pharmacokinetics. Further, we created a virtual patient population to account for the inter-patient variations seen in the trials. In each patient, we performed stochastic simulations and identified the VRC01 failure time following the model above. We compared the resulting distribution of failure times with those observed in the trials. We describe the methods we used here.

#### Data

We considered data of viral resurgence following VRC01 therapy during ATIs from two clinical trials, the A5340 trial and the NIH trial [17]. In the A5340 trial, 14 adult chronic HIV patients with a median ART duration of 4.7 years and viremia maintained below detection were administered 3 doses of VRC01 (40 mg/kg of body weight) intravenously, starting a week before the cessation of ART and with 3 week intervals. Plasma viremia was measured weekly to check for rebound (>20 copies/ml). Of the 14 participants, 1 participant stopped ART before VRC01 administration and was not part of the trial data analysis. We considered data from the remaining 13 patients. For each patient, we assumed the breakthrough time was anywhere within the week from the last undetectable to the first detectable viral load measurement.

The NIH trial had 10 participants with similar characteristics as the A5340 trial. The participants had been on ART much longer, however, with a median duration of 10 years, before entry into the trial. ART was stopped 3 days after the first VRC01 administration. VRC01 doses, at the same dosage as above, were administered subsequently on days 14 and 28 post ART cessation and once every month thereafter. Plasma viremia was measured every week for 1 month and then every 2 weeks until 6 months. The patients experienced virological breakthrough between 2 and 12 weeks from the discontinuation of ART. We considered this time of virological failure with uncertainties as defined above.

We note that both trials did not screen for pre-existing VRC01 resistance. While the primary endpoints of the trials were safety and tolerance, secondary endpoints were viral remission, based on which the trial data report Kaplan-Meier survival curves [17]. We considered the latter data too in our analysis.

#### VRC01 pharmacokinetics and pharmacodynamics

We let the efficacy of VRC01 against the drug-resistant mutant strain, *ε*_*m*_, be related to its plasma concentration, *A*, by the Hill function [51,57,99],

$$\varepsilon_{m}=\frac{A(t)}{IC_{50,m}+A(t)}$$ (17)

where *IC*_50,*m*_ is the value of *A* at which VRC01 is 50% efficacious. For simplicity, we set the Hill coefficient to 1. The antibody concentration has been observed to decline in a biphasic manner upon dosing [26]. We therefore described the time course of the antibody concentration using the following expression when successive doses are administered at time points *ω*_1_,*ω*_2_, and so on:

$$A(t)=\sum_{i=1}^{W}\left(A_{1}e^{-\eta_{1}(t-\omega_{i})}+A_{2}e^{-\eta_{2}(t-\omega_{i})}\right)\cdot H\left[t-\omega_{i}\right]$$ (18)

Here, *A*_1_ and *A*_2_ are the portions of a dose that decay in the first and second phases, respectively, with decay rates *η*_1_ and *η*_2_, and *W* is the total number of doses. We estimated the pharmacokinetic parameters using fits of the above equation to data of VRC01 concentrations following a single dose [26].

#### ART efficacy

For the duration that ART is used simultaneously with VRC01, we replaced the term (1−*ε*_*m*_) in Eqs 14 and 15 by 1−*ε*_*comb*_ = (1−*ε*_*m*_)(1−*ε*_*ART*_), where *ε*_*ART*_ is the efficacy of ART and *ε*_*comb*_ is the combined efficacy of VRC01 and ART. We let *ε*_*ART*_ be constant while on ART and set it to zero thereafter.

#### Virtual patient population

To capture inter-patient variations in the response to bNAb therapy, we constructed a virtual population of clinical trial participants as follows. For simplicity, we considered variations in two factors, the initial latent pool size, *L*_0_, and the viral production rate, *p*, across individuals. We assumed that variations in all host factors could be subsumed in the variation in *L*_0_ and that variations in viral factors could be subsumed in *p*. Introducing variations in other parameters did not affect our findings, as we found by varying the *IC*_50_ of VRC01 across patients (S5 Fig), justifying the latter assumption. We let *L*_0_ vary log-normally and *p* normally across individuals. Thus, the corresponding density functions were

$$f_{L_{0}}(l)=\frac{1}{l\sigma_{l}\sqrt{2\pi }}\exp\left(-\frac{\ln l-\mu_{l}}{2\sigma_{l}^{2}}\right)$$ (19)

and

$$f_{p}(\eta )=\frac{1}{\sigma_{p}\sqrt{2\pi }}\exp\left(-\frac{\eta -\mu_{p}}{2\sigma_{p}^{2}}\right)$$ (20)

where *μ*_*l*_ and *σ*_*l*_ are the mean and standard deviation of *lnL*_0_, and *μ*_*p*_ and *σ*_*p*_ are the mean and standard deviation of *p*, respectively. The parameters in the distributions were chosen based on earlier studies or to fit the results of the A5340 trial. From the resulting distributions, we sampled 10000 pairs of values of *L*_0_ and *p*, with each pair representing an individual. We thus created a virtual patient population, which we then subjected to VRC01 therapy according to the protocols in the respective trials. For each individual, we ran a stochastic simulation (Eqs 1–18) and examined the dynamics of virological breakthrough. From the resulting dynamics, we constructed the distribution of breakthrough times and used it to build Kaplan-Meier survival plots sampling virtual patients mimicking the clinical trial protocols.

## Supporting information

S1 Text VRC01 therapy failure is unlikely to be due to *de novo* mutation. (DOCX)

S2 Text MATLAB program used for simulations. (DOCX)

S1 Table Relative replicative fitness, relative infectivity, and pre-existing frequencies of various mutants. Replicative fitness was estimated by analyzing competitive growth assays where available (S1 Fig) and by assuming zero epistasis otherwise. For the N279K and N280D mutants, competitive growth assays indicated replicative fitness indistinguishable from the wild-type. The relative infectivity of each strain was calculated as the ratio of its % infectivity to that of the wild type, reported elsewhere [28]. The frequencies were obtained using our model (Eqs (1)–(10)).
(XLSX)

S1 Fig Estimation of fitness using data from competitive growth assays. We applied a previous formalism [59] to analyze competitive growth assays, where the selective advantage, *s*, of a mutant relative to the wild-type is given by *s* =ln[*H*(*t*)/*H*(0)]/ln[*W*(*t*)/*W*(0)]+*δt*], where *W*(*t*)/*W*(0) is the fold expansion of wild type virus at time *t*; *H*(*t*)/*H*(0) is the fold change of the mutant-to-wild type ratio at time *t*; and *δ* is the death rate of infected cells. Rewriting this equation as *Y* = *sX*, where *Y* = ln[*H*(*t*)/*H*(0)] and *X* = ln[*W*(*t*)/*W*(0)]+*δt*, we estimate *s* by fits (lines) to corresponding data (symbols) [28] of *Y* vs. *X* for three mutants: **(A)** R456W, **(B)** G458D, **(C)** N279K-N280D-R456W-G458D. The best-fit estimates (95%CI) of *s* are -0.568 (-0.6089, -0.5272) for R456W; -0.5091 (-0.5662, -0.452) for G458D; and -0.5672 (-0.6075, -0.5269) for N279K-N280D-R456W-G458D. The relative replicative fitness of the respective strains are obtained as *ζ* = (1+*s*).
(TIF)

S2 Fig VRC01 failure via *de novo* mutation. Predictions of stochastic simulations (Eqs. S1-S11), showing (A) time-evolution of the latent cell pool, (B) distribution of the waiting time for the reactivation from latency, (C) time-evolution of activated or productively infected cells, and (D) distribution of the waiting time for the first productively infected cell carrying a VRC01-resistant strain (bar graph). (In (A) and (C), the different lines represent different stochastic realizations.) Variation of the latter distribution is shown with (E) initial latent cell pool size (cells), (F) VRC01 efficacy, and (G) viral production rate (virions/cell/day). In (D)-(G), the corresponding probability density function calculated using the deterministic formalism (Eqs. S12-S17) is shown as solid lines. (H) Expected waiting time, τm, for the formation of a productively infected cell carrying a VRC01-resistant provirus, calculated using the deterministic formalism as a function of the initial latent pool size for the VRC01 efficacy indicated, which is the efficacy against the wild-type averaged over the dosing interval.
(TIF)

S3 Fig Estimation of inter-patient variation in latent pool size. Model predictions similar to those in **(A)** Fig 4C and **(B)** Fig 4D with virtual patient populations created by sampling *L*_0_ (cells) from the different distributions indicated. The red line in (B) is data from the A5340 trial.
(TIF)

S4 Fig Sensitivity to VRC01 *IC*_50_. Model predictions similar to those in (A) Fig 4C and (B) Fig 4D with different values of the *IC*_50_ (*μg/mL*) indicated. The red line in (B) is data from the A5340 trial.
(TIF)

S5 Fig Effect of inter-patient variation of VRC01 *IC*_50_. Model predictions similar to those in (A) Fig 4C and (B) Fig 4D but with the *IC*_50_ (instead of p) in each virtual patient drawn from a log-normal distribution (*Log*_10_*IC*_50_~*N*(2.86, 0.35)) (Inset). The red line in (B) is data from the A5340 trial.
(TIF)

S6 Fig Maximizing remission with VRC01. Heat map showing the median rebound time, corresponding to the detection limit of 20 copies/mL, for different VRC01 dosages and half-lives. For each parameter combination, a virtual population of 10000 individuals was employed following the dosing schedule in the A5340 schedule, as in Fig 4. Note that the modified bNAb VRC01LS has a >4-fold longer half-life than VRC01, and has been tested in healthy and uninfected adults for safety and pharmacokinetics [100]. The terminal half-life, *η*_1_, has been varied accordingly. The initial total antibody concentration (*A*_1_+*A*_2_) has been varied while keeping the ratio*A*_1_/*A*_2_ fixed, with the maximum (*A*_1_+*A*_2_) (in *μg/mL*) corresponding to the 40 mg/kg dosage of VRC01 (fitted from data [26]), which appears to be the maximum dosage of VRC01 reported. The other parameters are the same as in Fig 4.
(TIF)

## References

[1] Arts EJ, Hazuda DJ. HIV-1 Antiretroviral drug therapy. Cold Spring Harb Perspect Med. 2012;2:a007161 10.1101/cshperspect.a007161 22474613 PMC3312400

[2] Chun TW, Davey RT, Engel D, Lane HC, Fauci AS. AIDS: Re-emergence of HIV after stopping therapy. Nature. 1999;401:874–875. 10.1038/44755 10553903

[3] Chun TW, Moir S, Fauci AS. HIV reservoirs as obstacles and opportunities for an HIV cure. Nat Immunol. 2015;16:584–589. 10.1038/ni.3152 25990814

[4] Chun TW, Engel D, Berrey MM, Shea T, Corey L, Fauci AS. Early establishment of a pool of latently infected, resting CD4+ T cells during primary HIV-1 infection. Proc Natl Acad Sci U S A. 1998;95:8869–8873. 10.1073/pnas.95.15.8869 9671771 PMC21169

[5] Hosmane NN, Kwon KJ, Bruner KM, Capoferri AA, Beg S, Rosenbloom DIS, et al Proliferation of latently infected CD4+ T cells carrying replication-competent HIV-1: Potential role in latent reservoir dynamics. J Exp Med. 2017;214:959–972. 10.1084/jem.20170193 28341641 PMC5379987

[6] Reeves DB, Duke ER, Wagner TA, Palmer SE, Spivak AM, Schiffer JT. A majority of HIV persistence during antiretroviral therapy is due to infected cell proliferation. Nat Commun. 2018;9:4811 10.1038/s41467-018-06843-5 30446650 PMC6240116

[7] Finzi D, Blankson J, Siliciano JD, Margolick JB, Chadwick K, Pierson T, et al Latent infection of CD4+ T cells provides a mechanism for lifelong persistence of HIV-1, even in patients on effective combination therapy. Nat Med. 1999;5:512–517. 10.1038/8394 10229227

[8] Weinberger LS, Burnett JC, Toettcher JE, Arkin AP, Schaffer D V. Stochastic gene expression in a lentiviral positive-feedback loop: HIV-1 Tat fluctuations drive phenotypic diversity. Cell. 2005;122:169–182. 10.1016/j.cell.2005.06.006 16051143

[9] Ho Y-C, Shan L, Hosmane NN, Wang J, Laskey SB, Rosenbloom DIS, et al Replication-competent noninduced proviruses in the latent reservoir increase barrier to HIV-1 cure. Cell. 2013;155:540–551. 10.1016/j.cell.2013.09.020 24243014 PMC3896327

[10] Sáez-Cirión A, Bacchus C, Hocqueloux L, Avettand-Fenoel V, Girault I, Lecuroux C, et al Post-treatment HIV-1 controllers with a long-term virological remission after the interruption of early initiated antiretroviral therapy ANRS VISCONTI Study. PLoS Pathog. 2013;9:e1003211 10.1371/journal.ppat.1003211 23516360 PMC3597518

[11] Deeks SG, Lewin SR, Ross AL, Ananworanich J, Benkirane M, Cannon P, et al International AIDS Society global scientific strategy: towards an HIV cure 2016. Nat Med. 2016;22:839–850. 10.1038/nm.4108 27400264 PMC5322797

[12] Cockerham LR, Hatano H, Deeks SG. Post-treatment controllers: Role in HIV “cure” research. Curr HIV/AIDS Rep. 2016;13:1–9. 10.1007/s11904-016-0296-x 26781112

[13] Halper-Stromberg A, Nussenzweig MC. Towards HIV-1 remission: Potential roles for broadly neutralizing antibodies. J Clin Invest. 2016;126:415–423. 10.1172/JCI80561 26752643 PMC4731188

[14] Wen Y, Bar KJ, Li JZ. Lessons learned from HIV antiretroviral treatment interruption trials. Curr Opin HIV AIDS. 2018;13:416–421. 10.1097/COH.0000000000000484 29878912

[15] Zhou T, Georgiev I, Wu X, Yang ZY, Dai K, Finzi A, et al Structural basis for broad and potent neutralization of HIV-1 by antibody VRC01. Science. 2010;329:811–817. 10.1126/science.1192819 20616231 PMC2981354

[16] Wu X, Yang ZY, Li Y, Hogerkorp CM, Schief WR, Seaman MS, et al Rational design of envelope identifies broadly neutralizing human monoclonal antibodies to HIV-1. Science. 2010;329:856–861. 10.1126/science.1187659 20616233 PMC2965066

[17] Bar KJ, Sneller MC, Harrison LJ, Justement JS, Overton ET, Petrone ME, et al Effect of HIV antibody VRC01 on viral rebound after treatment interruption. N Engl J Med. 2016;375:2037–2050. 10.1056/NEJMoa1608243 27959728 PMC5292134

[18] Caskey M, Klein F, Lorenzi JCC, Seaman MS, West AP, Buckley N, et al Viraemia suppressed in HIV-1-infected humans by broadly neutralizing antibody 3BNC117. Nature. 2015;522:487–491. 10.1038/nature14411 25855300 PMC4890714

[19] Lu LL, Suscovich TJ, Fortune SM, Alter G. Beyond binding: antibody effector functions in infectious diseases. Nat Rev Immunol. 2018;18:46–61. 10.1038/nri.2017.106 29063907 PMC6369690

[20] Nishimura Y, Gautam R, Chun TW, Sadjadpour R, Foulds KE, Shingai M, et al Early antibody therapy can induce long-lasting immunity to SHIV. Nature. 2017;543:559–563. 10.1038/nature21435 28289286 PMC5458531

[21] Borducchi EN, Liu J, Nkolola JP, Cadena AM, Yu WH, Fischinger S, et al Antibody and TLR7 agonist delay viral rebound in SHIV-infected monkeys. Nature. 2018;563:360–364. 10.1038/s41586-018-0600-6 30283138 PMC6237629

[22] Rudicell RS, Kwon YD, Ko S-Y, Pegu A, Louder MK, Georgiev IS, et al Enhanced potency of a broadly neutralizing HIV-1 antibody in vitro improves protection against lentiviral infection in vivo. J Virol. 2014;88:12669–12682. 10.1128/JVI.02213-14 25142607 PMC4248941

[23] Li JZ, Etemad B, Ahmed H, Aga E, Bosch RJ, Mellors JW, et al The size of the expressed HIV reservoir predicts timing of viral rebound after treatment interruption. AIDS. 2016;30:343–353. 10.1097/QAD.0000000000000953 26588174 PMC4840470

[24] Morris L, Mkhize NN. Prospects for passive immunity to prevent HIV infection. PLoS Med. 2017;14:e1002436 10.1371/journal.pmed.1002436 29136030 PMC5685477

[25] Reeves DB, Huang Y, Duke ER, Mayer BT, Cardozo-Ojeda EF, Boshier FA, et al Mathematical modeling to reveal breakthrough mechanisms in the HIV Antibody Mediated Prevention (AMP) trials. PLoS Comput Biol. 2020;16:e1007626 10.1371/journal.pcbi.1007626 32084132 PMC7055956

[26] Lynch RM, Boritz E, Coates EE, DeZure A, Madden P, Costner P, et al Virologic effects of broadly neutralizing antibody VRC01 administration during chronic HIV-1 infection. Sci Transl Med. 2015;7:319ra206 10.1126/scitranslmed.aad5752 26702094 PMC12366723

[27] Mayer KH, Seaton KE, Huang Y, Grunenberg N, Isaacs A, Allen M, et al Safety, pharmacokinetics, and immunological activities of multiple intravenous or subcutaneous doses of an anti-HIV monoclonal antibody, VRC01, administered to HIV-uninfected adults: Results of a phase 1 randomized trial. PLoS Med. 2017;14:e1002435 10.1371/journal.pmed.1002435 29136037 PMC5685476

[28] Lynch RM, Wong P, Tran L, O’Dell S, Nason MC, Li Y, et al HIV-1 Fitness cost associated with escape from the VRC01 class of CD4 binding site neutralizing antibodies. J Virol. 2015;89:4201–4213. 10.1128/JVI.03608-14 25631091 PMC4442379

[29] Otsuka Y, Schmitt K, Quinlan BD, Gardner MR, Alfant B, Reich A, et al Diverse pathways of escape from all well-characterized VRC01-class broadly neutralizing HIV-1 antibodies. PLoS Pathog. 2018;14:e1007238 10.1371/journal.ppat.1007238 30125330 PMC6117093

[30] Tachibana S, Sasaki M, Tanaka T, Inoue M, Ophinni Y, Kotaki T, et al A 2-4-amino acid deletion in the V5 region of HIV-1 Env gp120 confers viral resistance to the broadly neutralizing human monoclonal antibody, VRC01. AIDS Res Hum Retroviruses. 2017;33:1248–1257. 10.1089/aid.2017.0063 28903577

[31] Wang W, Zirkle B, Nie J, Ma J, Gao K, Chen XS, et al N463 glycosylation site on V5 loop of a mutant gp120 regulates the sensitivity of HIV-1 to neutralizing monoclonal antibodies VRC01/03. J Acquir Immune Defic Syndr. 2015;69:270–277. 10.1097/QAI.0000000000000595 25751231 PMC4506726

[32] Van Laethem K, Theys K, Vandamme A-M. HIV-1 genotypic drug resistance testing: digging deep, reaching wide? Curr Opin Virol. 2015;14:16–23. 10.1016/j.coviro.2015.06.001 26114581

[33] Bruner KM, Wang Z, Simonetti FR, Bender AM, Kwon KJ, Sengupta S, et al A quantitative approach for measuring the reservoir of latent HIV-1 proviruses. Nature. 2019;566:120–125. 10.1038/s41586-019-0898-8 30700913 PMC6447073

[34] Silver N, Paynter M, McAllister G, Atchley M, Sayir C, Short J, et al Characterization of minority HIV-1 drug resistant variants in the United Kingdom following the verification of a deep sequencing-based HIV-1 genotyping and tropism assay. AIDS Res Ther. 2018;15:18 10.1186/s12981-018-0206-y 30409215 PMC6223033

[35] Estes JD, Kityo C, Ssali F, Swainson L, Makamdop KN, Del Prete GQ, et al Defining total-body AIDS-virus burden with implications for curative strategies. Nat Med. 2017;23:1271–1276. 10.1038/nm.4411 28967921 PMC5831193

[36] Salantes DB, Zheng Y, Mampe F, Srivastava T, Beg S, Lai J, et al HIV-1 latent reservoir size and diversity are stable following brief treatment interruption. J Clin Invest. 2018;128:3102–3115. 10.1172/JCI120194 29911997 PMC6026010

[37] Conway JM, Coombs D. A stochastic model of latently infected cell reactivation and viral blip generation in treated HIV patients. PLoS Comput Biol. 2011;7:1–25. 10.1371/journal.pcbi.1002033 21552334 PMC3084212

[38] Hill AL. Modeling HIV persistence and cure studies. Curr Opin HIV AIDS. 2018;13:428–434. 10.1097/COH.0000000000000490 29916846 PMC6112843

[39] Conway JM, Perelson AS. Post-treatment control of HIV infection. Proc Natl Acad Sci U S A. 2015;112:5467–5472. 10.1073/pnas.1419162112 25870266 PMC4418889

[40] Dar RD, Hosmane NN, Arkin MR, Siliciano RF, Weinberger LS. Screening for noise in gene expression identifies drug synergies. Science. 2014;344:1392–1396. 10.1126/science.1250220 24903562 PMC4122234

[41] Hill AL, Rosenbloom DIS, Fu F, Nowak MA, Siliciano RF. Predicting the outcomes of treatment to eradicate the latent reservoir for HIV-1. Proc Natl Acad Sci U S A. 2014;111:13475–13480. 10.1073/pnas.1406663111 25097264 PMC4169952

[42] Rong L, Perelson AS. Modeling latently infected cell activation: Viral and latent reservoir persistence, and viral blips in HIV-infected patients on potent therapy. PLoS Comput Biol. 2009;5:e1000533 10.1371/journal.pcbi.1000533 19834532 PMC2752194

[43] Conway JM, Perelson AS, Li JZ. Predictions of time to HIV viral rebound following ART suspension that incorporate personal biomarkers. PLoS Comput Biol. 2019;15:e1007229 10.1371/journal.pcbi.1007229 31339888 PMC6682162

[44] Pinkevych M, Cromer D, Tolstrup M, Grimm AJ, Cooper DA, Lewin SR, et al HIV Reactivation from latency after treatment interruption occurs on average every 5–8 days—Implications for HIV remission. PLoS Pathog. 2015;11:e1005000 10.1371/journal.ppat.1005000 26133551 PMC4489624

[45] Gupta V, Dixit NM. Trade-off between synergy and efficacy in combinations of HIV-1 latency-reversing agents. PLoS Comput Biol. 2018;14:e1006004 10.1371/journal.pcbi.1006004 29451894 PMC5833289

[46] Althaus CL, De Boer RJ. Intracellular transactivation of HIV can account for the decelerating decay of virus load during drug therapy. Mol Syst Biol. 2010;6:1–8. 10.1038/msb.2010.4 20160709 PMC2835566

[47] Rouzine IM, Weinberger AD, Weinberger LS. An evolutionary role for HIV latency in enhancing viral transmission. Cell. 2015;160:1002–1012. 10.1016/j.cell.2015.02.017 25723173 PMC4488136

[48] Davenport MP, Khoury DS, Cromer D, Lewin SR, Kelleher AD, Kent SJ. Functional cure of HIV: the scale of the challenge. Nat Rev Immunol. 2019;19:45–54. 10.1038/s41577-018-0085-4 30410126

[49] Ribeiro RM, Bonhoeffer S, Nowak MA. The frequency of resistant mutant virus before antiviral therapy. AIDS. 1998;12:461–465. 10.1097/00002030-199805000-00006 9543443

[50] Ribeiro RM, Bonhoeffer S. Production of resistant HIV mutants during antiretroviral therapy. Proc Natl Acad Sci U S A. 2000;97:7681–7686. 10.1073/pnas.97.14.7681 10884399 PMC16603

[51] Arora P, Dixit NM. Timing the emergence of resistance to anti-HIV drugs with large genetic barriers. PLoS Comput Biol. 2009;5:e1000305 10.1371/journal.pcbi.1000305 19282958 PMC2643484

[52] Gadhamsetty S, Dixit NM. Estimating frequencies of minority nevirapine-resistant strains in chronically HIV-1-infected individuals naive to nevirapine by using stochastic simulations and a mathematical model. J Virol. 2010;84:10230–10240. 10.1128/JVI.01010-10 20668070 PMC2937761

[53] Pennings PS. Standing genetic variation and the evolution of drug resistance in HIV. PLoS Comput Biol. 2012;8:e1002527 10.1371/journal.pcbi.1002527 22685388 PMC3369920

[54] Tripathi K, Balagam R, Vishnoi NK, Dixit NM. Stochastic simulations suggest that HIV-1 survives close to its error threshold. PLoS Comput Biol. 2012;8:e1002684 10.1371/journal.pcbi.1002684 23028282 PMC3441496

[55] Rosenbloom DIS, Hill AL, Rabi SA, Siliciano RF, Nowak MA. Antiretroviral dynamics determines HIV evolution and predicts therapy outcome. Nat Med. 2012;18:1378–1385. 10.1038/nm.2892 22941277 PMC3490032

[56] Hinkley T, Martins J, Chappey C, Haddad M, Stawiski E, Whitcomb JM, et al A systems analysis of mutational effects in HIV-1 protease and reverse transcriptase. Nat Genet. 2011;43:487–489. 10.1038/ng.795 21441930

[57] Brandenberg OF, Magnus C, Rusert P, Günthard HF, Regoes RR, Trkola A. Predicting HIV-1 transmission and antibody neutralization efficacy in vivo from stoichiometric parameters. PLoS Pathog. 2017;13:e1006313 10.1371/journal.ppat.1006313 28472201 PMC5417720

[58] Shet A, Nagaraja P, Dixit NM. Viral decay dynamics and mathematical modeling of treatment response. J Acquir Immune Defic Syndr. 2016;73:245–251. 10.1097/QAI.0000000000001101 27273158 PMC5172519

[59] Maree AFM, Keulen W, Boucher CAB, De Boer RJ. Estimating relative fitness in viral competition experiments. J Virol. 2000;74:11067–11072. 10.1128/jvi.74.23.11067-11072.2000 11070001 PMC113186

[60] Haddox HK, Dingens AS, Bloom JD. Experimental estimation of the effects of all amino-acid mutations to HIV’s envelope protein on viral replication in cell culture. PLoS Pathog. 2016;12:e1006114 10.1371/journal.ppat.1006114 27959955 PMC5189966

[61] Brooks K, Jones BR, Dilernia DA, Wilkins DJ, Claiborne DT, McInally S, et al HIV-1 variants are archived throughout infection and persist in the reservoir. PLoS Pathog. 2020;16:e1008378 10.1371/journal.ppat.1008378 32492044 PMC7295247

[62] Kepler TB, Perelson AS. Drug concentration heterogeneity facilitates the evolution of drug resistance. Proc Natl Acad Sci. 1998;95:11514–11519. 10.1073/pnas.95.20.11514 9751697 PMC21672

[63] Goulder P, Deeks SG. HIV control: Is getting there the same as staying there? PLoS Pathog. 2018;14:e1007222 10.1371/journal.ppat.1007222 30383857 PMC6211749

[64] Fraser C, Lythgoe K, Leventhal GE, Shirreff G, Hollingsworth TD, Alizon S, et al Virulence and Pathogenesis of HIV-1 Infection: An Evolutionary Perspective. Science. 2014;343:1243727–1243727. 10.1126/science.1243727 24653038 PMC5034889

[65] Prague M, Commenges D, Guedj J, Drylewicz J, Thiébaut R. NIMROD: A program for inference via a normal approximation of the posterior in models with random effects based on ordinary differential equations. Comput Methods Programs Biomed. 2013;111:447–458. 10.1016/j.cmpb.2013.04.014 23764196

[66] Lavielle M. Mixed Effects Models for the Population Approach. Chapman and Hall/CRC; 2014 10.1201/b17203

[67] van Gils MJ, Sanders RW. In vivo protection by broadly neutralizing HIV antibodies. Trends Microbiol. 2014;22:550–551. 10.1016/j.tim.2014.08.006 25169020

[68] de Bree GJ, Sanders RW. Broadly neutralising antibodies in post-treatment control. Lancet HIV. 2019;6:e271–e272. 10.1016/S2352-3018(19)30075-X 31000478

[69] Haynes BF, Burton DR, Mascola JR. Multiple roles for HIV broadly neutralizing antibodies. Sci Transl Med. 2019;11:eaaz2686 10.1126/scitranslmed.aaz2686 31666399 PMC7171597

[70] Schommers P, Gruell H, Abernathy ME, Tran M-K, Dingens AS, Gristick HB, et al Restriction of HIV-1 escape by a highly broad and potent neutralizing antibody. Cell. 2020;180:471–489.e22. 10.1016/j.cell.2020.01.010 32004464 PMC7042716

[71] Keele BF, Giorgi EE, Salazar-Gonzalez JF, Decker JM, Pham KT, Salazar MG, et al Identification and characterization of transmitted and early founder virus envelopes in primary HIV-1 infection. Proc Natl Acad Sci. 2008;105:7552–7557. 10.1073/pnas.0802203105 18490657 PMC2387184

[72] Shankarappa R, Margolick JB, Gange SJ, Rodrigo AG, Upchurch D, Farzadegan H, et al Consistent viral evolutionary changes associated with the progression of human immunodeficiency virus type 1 infection. J Virol. 1999;73:10489–10502. 10.1128/JVI.73.12.10489-10502.1999 10559367 PMC113104

[73] Crowell TA, Colby DJ, Pinyakorn S, Sacdalan C, Pagliuzza A, Intasan J, et al Safety and efficacy of VRC01 broadly neutralising antibodies in adults with acutely treated HIV (RV397): a phase 2, randomised, double-blind, placebo-controlled trial. Lancet HIV. 2019;6:e297–e306. 10.1016/S2352-3018(19)30053-0 31000477 PMC6693657

[74] Hashimoto M, Kamphorst AO, Im SJ, Kissick HT, Pillai RN, Ramalingam SS, et al CD8 T cell exhaustion in chronic infection and cancer: Opportunities for interventions. Annu Rev Med. 2018;69:301–318. 10.1146/annurev-med-012017-043208 29414259

[75] McLane LM, Abdel-Hakeem MS, Wherry EJ. CD8 T cell exhaustion during chronic viral infection and cancer. Annu Rev Immunol. 2019;37:457–495. 10.1146/annurev-immunol-041015-055318 30676822

[76] Johnson PLF, Kochin BF, McAfee MS, Stromnes IM, Regoes RR, Ahmed R, et al Vaccination alters the balance between protective immunity, exhaustion, escape, and death in chronic infections. J Virol. 2011 10.1128/jvi.00166-11 21411537 PMC3094965

[77] Baral S, Roy R, Dixit NM. Modeling how reversal of immune exhaustion elicits cure of chronic hepatitis C after the end of treatment with direct-acting antiviral agents. Immunol Cell Biol. 2018;96:969–980. 10.1111/imcb.12161 29744934 PMC6220890

[78] Baral S, Antia R, Dixit NM. A dynamical motif comprising the interactions between antigens and CD8 T cells may underlie the outcomes of viral infections. Proc Natl Acad Sci. 2019;116:17393–17398. 10.1073/pnas.1902178116 31413198 PMC6717250

[79] Baral S, Raja R, Sen P, Dixit NM. Towards multiscale modeling of the CD8 + T cell response to viral infections. Wiley Interdiscip Rev Syst Biol Med. 2019;11:e1446 10.1002/wsbm.1446 30811096 PMC6614031

[80] Wu X, Zhou T, Zhu J, Zhang B, Georgiev I, Wang C, et al Focused evolution of HIV-1 neutralizing antibodies revealed by structures and deep sequencing. Science. 2011;333:1593–1602. 10.1126/science.1207532 21835983 PMC3516815

[81] Wu X, Zhang Z, Schramm CA, Joyce MG, Do Kwon Y, Zhou T, et al Maturation and diversity of the VRC01-antibody lineage over 15 years of chronic HIV-1 infection. Cell. 2015;161:470–485. 10.1016/j.cell.2015.03.004 25865483 PMC4706178

[82] Scheid JF, Mouquet H, Ueberheide B, Diskin R, Klein F, Oliveira TYK, et al Sequence and structural convergence of broad and potent HIV antibodies that mimic CD4 binding. Science. 2011;333:1633–1637. 10.1126/science.1207227 21764753 PMC3351836

[83] Gilbert PB, Juraska M, DeCamp AC, Karuna S, Edupuganti S, Mgodi N, et al Basis and statistical design of the passive HIV-1 antibody mediated prevention (AMP) test-of-concept efficacy trials. Stat Commun Infect Dis. 2017;9 10.1515/scid-2016-0001 29218117 PMC5714515

[84] Huang Y, Karuna S, Carpp LN, Reeves D, Pegu A, Seaton K, et al Modeling cumulative overall prevention efficacy for the VRC01 phase 2b efficacy trials. Hum Vaccines Immunother. 2018;14:2116–2127. 10.1080/21645515.2018.1462640 29683765 PMC6183277

[85] Raja R, Pareek A, Newar K, Dixit NM. Mutational pathway maps and founder effects define the within-host spectrum of hepatitis C virus mutants resistant to drugs. PLoS Pathog. 2019;15:e1007701 10.1371/journal.ppat.1007701 30934020 PMC6459561

[86] Prague M, Gerold JM, Balelli I, Pasin C, Li JZ, Barouch DH, et al Viral rebound kinetics following single and combination immunotherapy for HIV / SIV. bioRxiv. 2019; 1–72. 10.1101/700401

[87] Desikan R, Raja R, Dixit NM. Early exposure to broadly neutralizing antibodies may trigger a dynamical switch from progressive disease to lasting control of SHIV infection. PLoS Comput Biol. 2020; 16: e1008064 10.1371/journal.pcbi.1008064 32817614 PMC7462315

[88] Cardozo EF, Andrade A, Mellors JW, Kuritzkes DR, Perelson AS, Ribeiro RM. Treatment with integrase inhibitor suggests a new interpretation of HIV RNA decay curves that reveals a subset of cells with slow integration. PLoS Pathog. 2017;13:e1006478 10.1371/journal.ppat.1006478 28678879 PMC5513547

[89] Schoofs T, Klein F, Braunschweig M, Kreider EF, Feldmann A, Nogueira L, et al HIV-1 therapy with monoclonal antibody 3BNC117 elicits host immune responses against HIV-1. Science. 2016;352:997–1001. 10.1126/science.aaf0972 27199429 PMC5151174

[90] Garg AK, Desikan R, Dixit NM. Preferential presentation of high-affinity immune complexes in germinal centers can explain how passive immunization improves the humoral response. Cell Rep. 2019;29:3946–3957.e5. 10.1016/j.celrep.2019.11.030 31851925 PMC7116025

[91] DiLillo DJ, Ravetch J V. Differential Fc-Receptor Engagement Drives an Anti-tumor Vaccinal Effect. Cell. 2015;161:1035–1045. 10.1016/j.cell.2015.04.016 25976835 PMC4441863

[92] Chen BK, Gandhi RT, Baltimore D. CD4 down-modulation during infection of human T cells with human immunodeficiency virus type 1 involves independent activities of vpu, env, and nef. J Virol. 1996. 10.1128/jvi.70.9.6044-6053.1996 PMC190625 8709227

[93] Dixit NM, Perelson AS. HIV dynamics with multiple infections of target cells. Proc Natl Acad Sci. 2005;102:8198–8203. 10.1073/pnas.0407498102 15928092 PMC1149399

[94] Josefsson L, King MS, Makitalo B, Brannstrom J, Shao W, Maldarelli F, et al Majority of CD4+ T cells from peripheral blood of HIV-1-infected individuals contain only one HIV DNA molecule. Proc Natl Acad Sci. 2011;108:11199–11204. 10.1073/pnas.1107729108 21690402 PMC3131354

[95] Nagaraja P, Alexander HK, Bonhoeffer S, Dixit NM. Influence of recombination on acquisition and reversion of immune escape and compensatory mutations in HIV-1. Epidemics. 2016;14:11–25. 10.1016/j.epidem.2015.09.001 26972510

[96] Suryavanshi GW, Dixit NM. Emergence of recombinant forms of HIV: Dynamics and scaling. PLoS Comput Biol. 2007;3:e205 10.1371/journal.pcbi.0030205 17967052 PMC2041978

[97] Ramratnam B, Bonhoeffer S, Binley J, Hurley A, Zhang L, Mittler JE, et al Rapid production and clearance of HIV-1 and hepatitis C virus assessed by large volume plasma apheresis. Lancet. 1999;354:1782–1785. 10.1016/S0140-6736(99)02035-8 10577640

[98] Gillespie DT. Exact stochastic simulation of coupled chemical reactions. J Phys Chem. 1977;81:2340–2361. 10.1021/j100540a008

[99] Dixit NM, Perelson AS. Complex patterns of viral load decay under antiretroviral therapy: influence of pharmacokinetics and intracellular delay. J Theor Biol. 2004;226:95–109. 10.1016/j.jtbi.2003.09.002 14637059

[100] Gaudinski MR, Coates EE, Houser K V., Chen GL, Yamshchikov G, Saunders JG, et al Safety and pharmacokinetics of the Fc-modified HIV-1 human monoclonal antibody VRC01LS: A Phase 1 open-label clinical trial in healthy adults. PLoS Med. 2018;15:e1002493 10.1371/journal.pmed.1002493 29364886 PMC5783347

