# Supplementary material for: Pre-existing resistance in the latent reservoir can compromise VRC01 therapy during chronic HIV-1 infection
Source: PLoS Comput Biol. 2020 Nov 30;16(11):e1008434. doi: 10.1371/journal.pcbi.1008434 (PMC7728175; doi:10.1371/journal.pcbi.1008434)
Supplement: S2 Text — (DOCX) [file pcbi.1008434.s002.docx]

**S2 Text. MATLAB program used for simulations**

p = 4.2*10^(-3); % Latent cell proliferation rate

ac = 7.8*10^(-4); % Latent cell activation rate

dl= 0.004; % latent cell death rate

it = 50; % Number of realizations

tfinal = 400; % Final time point for each realization

Time=0:0.1:tfinal; % Creating a vector of time intervals from 0 to tfinal

k0 = 1.6*10^(-12); % Infectivity of the viral strain

U = 15*10^(9); % Uninfected cell population

c = 23; % Death rate of virus

delta = 1; % Death rate of infected cell

mu = 3E-5; % Mutation rate

Ab01=450.6;Ab02 = 1253; % Initial antibody concentration

n1 = -0.09332; n2 = -1.374; % Antibody decay rate

IC50 = 800; % IC50 of the rebound strain

fit = 0.86; % Fitness of the rebound strain

f = 10^(-6); % fraction of new infections producing latent cells

i = 1;

A1 = NaN(it,length(Time),2);V1 = NaN(it,length(Time));

WT = NaN(it,1);

%Normal distribution of p

sN = 200;

mN = 2100;

N = sN.*randn(it,1) + mN;

X = (k0*N*delta*fit*U)/c;

%Log normal distribution of initial latent pool

mL = 14.5;

sL = 1;

Lat=lognrnd(mL,sL,it,1);

L_init = 0.000227*Lat;

Ac_init = 0; M = zeros(it,1);

T1=0;T2=21;T3 = 42; %VRC01 infusion protocol according to A5340 trial

e_ART = 0.99;T_tostopART = 7; %ART efficacy and time to stop ART after VRC01 first infusion

parfor k=1:1:it

k

i = 1;

A =[];

t = [];

Ab = [];

e = [];

V = [];

index = [];

ans3 = [];

ans4 = [];

A(1,1) = L_init(k); A(1,2) = Ac_init;

t(1) =0; Ab(1) = (Ab01+Ab02); e(1) = 1-((1-(Ab(1)/(Ab(1) + IC50)))*(1-e_ART));

t(1) =0;

Aint = NaN(length(Time),2);Vint = NaN(length(Time),1);

while(t(i) < tfinal && i<= 340000)

L = A(i,1); Ac = A(i,2); V(1) = (N(k)*fit*delta*A(1,2))/(15000*c);

r = [];

%Reaction matrix for Gillespie

r(1) = p*L;

r(2) = dl*L;

r(3) = ac*L;

r(4) = X(k)*f*(1-mu)*(1-e(i))*Ac;

r(5) = X(k)*(1-f)*(1-mu)*(1-e(i))*Ac;

r(6) = delta*Ac;

R = sum(r);

random = rand(1);

t(i+1) = t(i) + (1/R)*log(1/random); %Generating next time step for a reaction to happen

% Calculating time dependent antibody concentration and antibody efficacy

if t(i+1) < T1

Ab(i+1) = 0;

elseif t(i+1) >= T1 && t(i+1) < T2

Ab(i+1) = (Ab01*exp(n1*(t(i+1) -T1))+Ab02*exp(n2*(t(i+1) -T1)));

elseif t(i+1) >= T2 && t(i+1)< T3

Ab(i+1) = (Ab01*exp(n1*(t(i+1) -T1))+Ab02*exp(n2*(t(i+1) -T1))+Ab01*exp(n1*(t(i+1) -T2)) + Ab02*exp(n2*(t(i+1) -T2)));

elseif t(i+1) >= T3

Ab(i+1) = (Ab01*exp(n1*(t(i+1) -T1))+Ab02*exp(n2*(t(i+1) -T1))+Ab01*exp(n1*(t(i+1) -T2)) + Ab02*exp(n2*(t(i+1) -T2)) + Ab01*exp(n1*(t(i+1) -T3))+Ab02*exp(n2*(t(i+1) -T3)));

end

if t(i+1) <= T_tostopART

e(i+1) = 1-((1-((Ab(i+1))^1/((Ab(i+1))^1 + IC50^1)))*(1-e_ART));

else

e(i+1) = (Ab(i+1))^1/((Ab(i+1))^1 +IC50^1);

end

random2 = rand(1);

ran = R*random2;

reac = [ 1 0;

-1 0;

-1 1;

1 0;

0 1;

0 -1];

CumRate=cumsum(r);

for a=1:6

if((CumRate(a)-ran)>0)

chrxn=a;

A(i+1,1) = A(i,1) + reac(chrxn,1);

A(i+1,2) = A(i,2) + reac(chrxn,2);

break;

end

end

i = i+1;

V(i) = (N(k)*fit*delta*A(i,2))/(15000*c); %Viral load from pseudo steady state assumption

end

[t, index] = unique(t);

Aint(:,1)= interp1(t,A(index,1),Time); % Interpolation

Aint(:,2)= interp1(t,A(index,2),Time);

Vint(:) = interp1(t,V(index),Time);

A1(k,:,:)=Aint(:,:);

V1(k,:) = Vint(:);

M(k) = max(A(:,2));

ans1 = find(A(:,2),1);

ans2 = isempty(ans1);

if ans2 == 0

WT(k) = t(ans1); % Waiting time for the first reactivated cell

end

ans3 = find(V(:)>=20,1);

ans4 = isempty(ans3);

if ans4 == 0

RT(k) = t(ans3);

virus_RT(k)=V(ans3); % Time to reach viral load of 20 copies/mL

end

ans7 = find(V(:)>=200,1);

ans8 = isempty(ans7);

if ans8 == 0

RTint(k) = t(ans7);

virus_int_RT(k)=V(ans7); % Time to reach viral load of 200 copies/mL

end

end

WT(isnan(WT))=0;

WT(WT==0) = [];

AvgWT = mean(WT);

RT(isnan(RT))=0;

RT(RT==0) = [];

AvgRT = mean(RT);

S_RT = std(RT);

RTint(isnan(RTint))=0;

RTint(RTint==0) = [];

AvgRTint = mean(RTint);
