# Supplementary figures and images for: Pre-existing resistance in the latent reservoir can compromise VRC01 therapy during chronic HIV-1 infection
Source: PLoS Comput Biol. 2020 Nov 30;16(11):e1008434. doi: 10.1371/journal.pcbi.1008434 (PMC7728175; doi:10.1371/journal.pcbi.1008434)

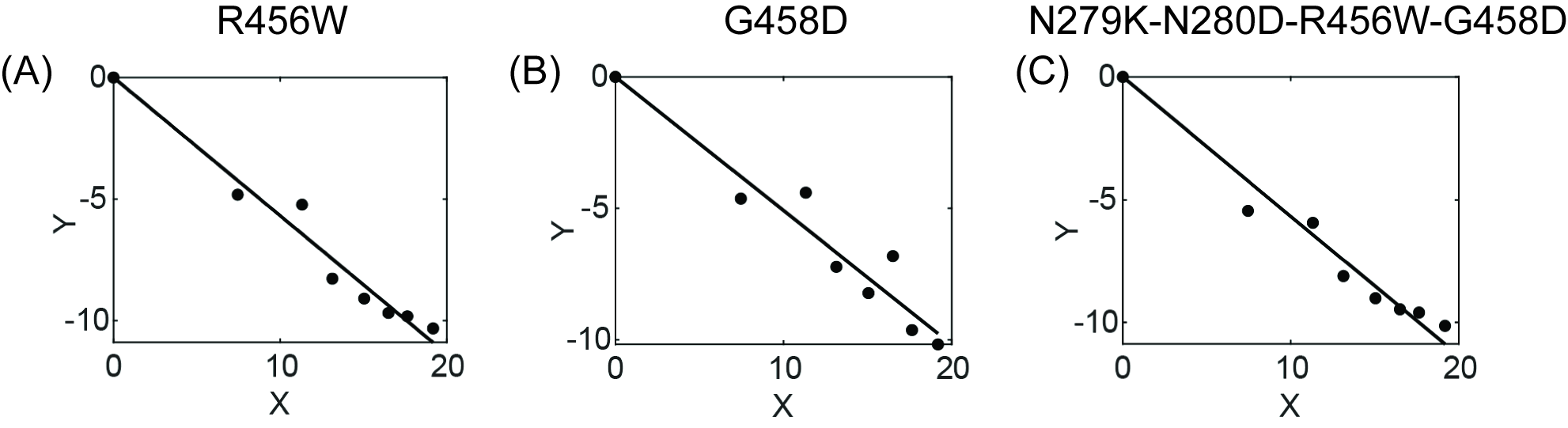

Supplement: S1 Fig — We applied a previous formalism [59] to analyze competitive growth assays, where the selective advantage, s, of a mutant relative to the wild-type is given by s =ln[H(t)/H(0)]/ln[W(t)/W(0)]+δt], where W(t)/W(0) is the fold expansion of wild type virus at time t; H(t)/H(0) is the fold change of the mutant-to-wild type ratio at time t; and δ is the death rate of infected cells. Rewriting this equation as Y = sX, where Y = ln[H(t)/H(0)] and X = ln[W(t)/W(0)]+δt, we estimate s by fits (lines) to corresponding data (symbols) [28] of Y vs. X for three mutants: (A) R456W, (B) G458D, (C) N279K-N280D-R456W-G458D. The best-fit estimates (95%CI) of s are -0.568 (-0.6089, -0.5272) for R456W; -0.5091 (-0.5662, -0.452) for G458D; and -0.5672 (-0.6075, -0.5269) for N279K-N280D-R456W-G458D. The relative replicative fitness of the respective strains are obtained as ζ = (1+s). (TIF) [file pcbi.1008434.s004.tif]

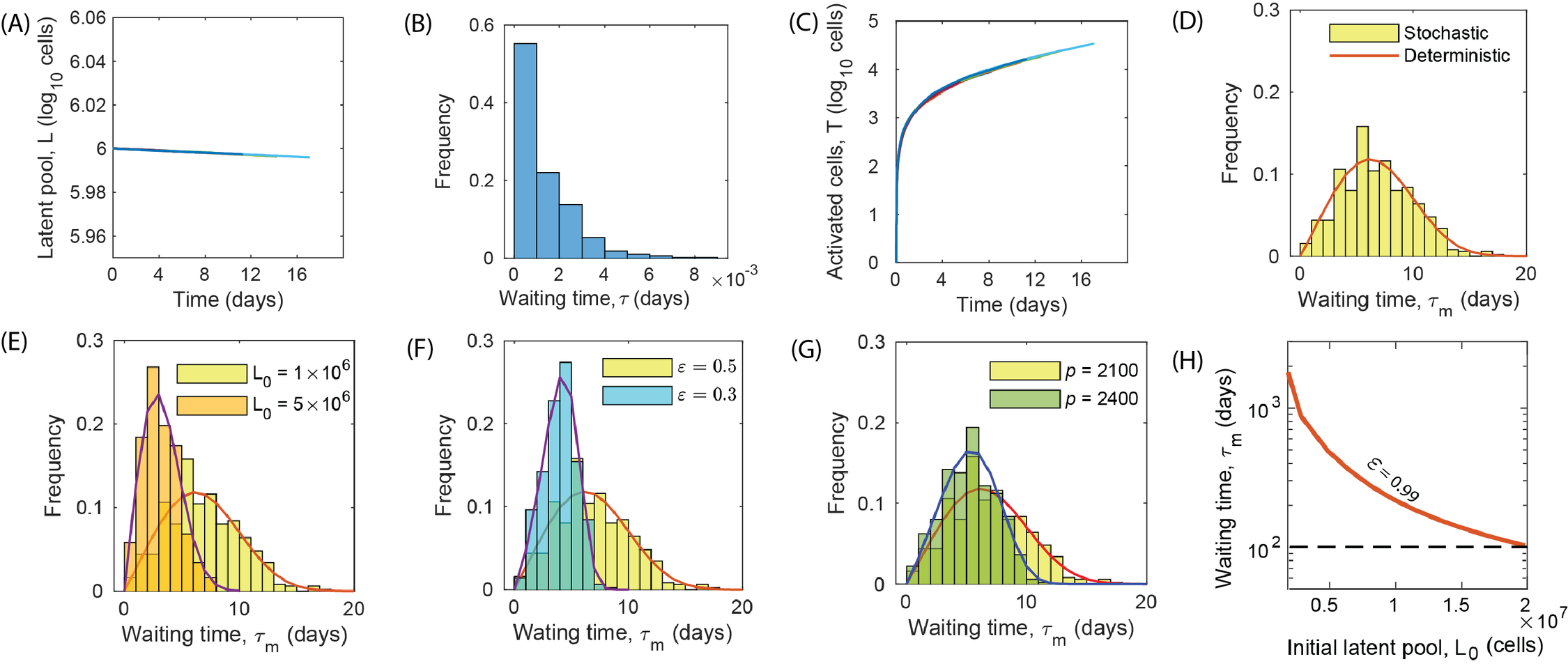

Supplement: S2 Fig — Predictions of stochastic simulations (Eqs. S1-S11), showing (A) time-evolution of the latent cell pool, (B) distribution of the waiting time for the reactivation from latency, (C) time-evolution of activated or productively infected cells, and (D) distribution of the waiting time for the first productively infected cell carrying a VRC01-resistant strain (bar graph). (In (A) and (C), the different lines represent different stochastic realizations.) Variation of the latter distribution is shown with (E) initial latent cell pool size (cells), (F) VRC01 efficacy, and (G) viral production rate (virions/cell/day). In (D)-(G), the corresponding probability density function calculated using the deterministic formalism (Eqs. S12-S17) is shown as solid lines. (H) Expected waiting time, τm, for the formation of a productively infected cell carrying a VRC01-resistant provirus, calculated using the deterministic formalism as a function of the initial latent pool size for the VRC01 efficacy indicated, which is the efficacy against the wild-type averaged over the dosing interval. (TIF) [file pcbi.1008434.s005.tif]

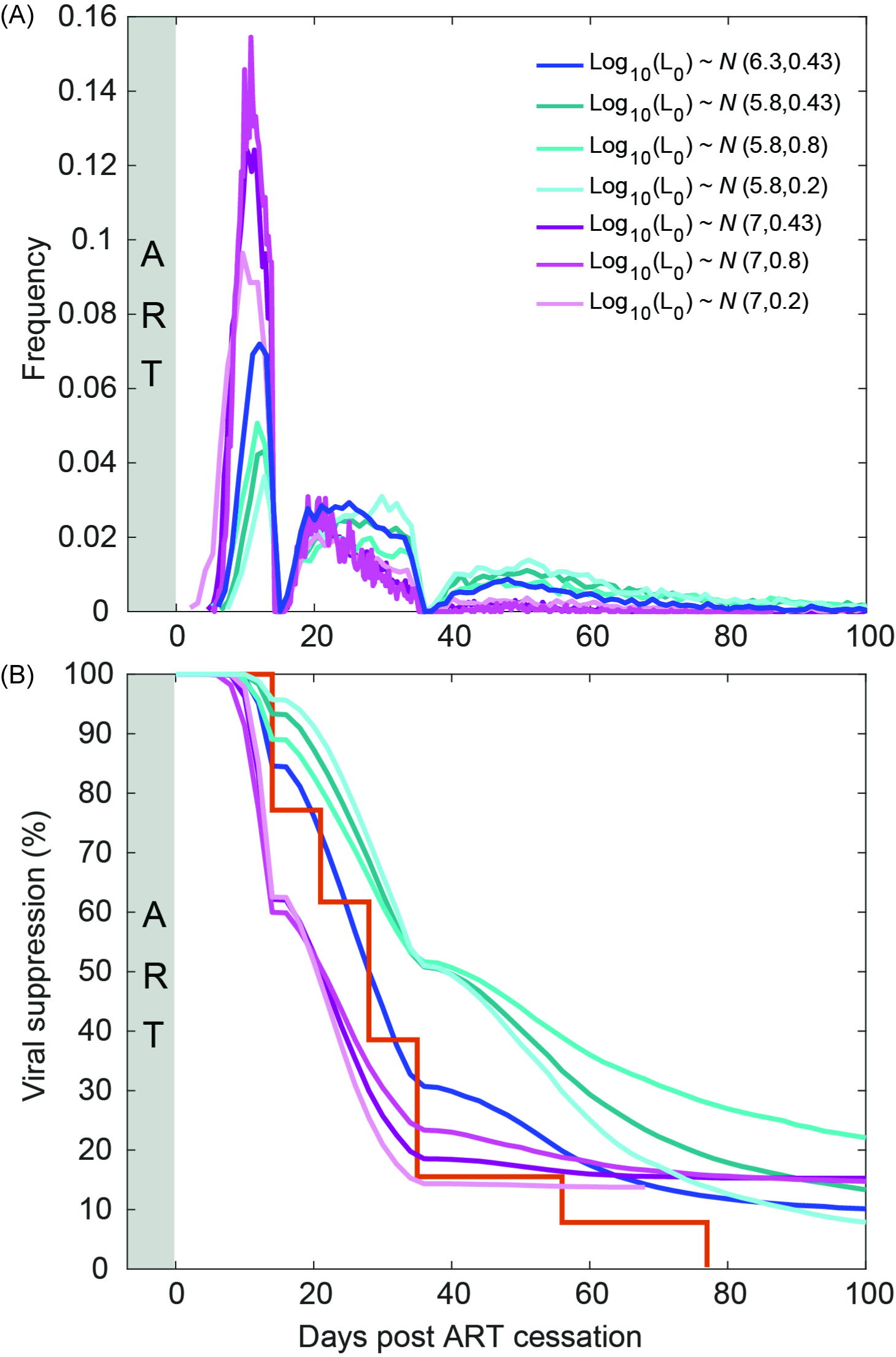

Supplement: S3 Fig — Model predictions similar to those in (A) Fig 4C and (B) Fig 4D with virtual patient populations created by sampling L0 (cells) from the different distributions indicated. The red line in (B) is data from the A5340 trial. (TIF) [file pcbi.1008434.s006.tif]

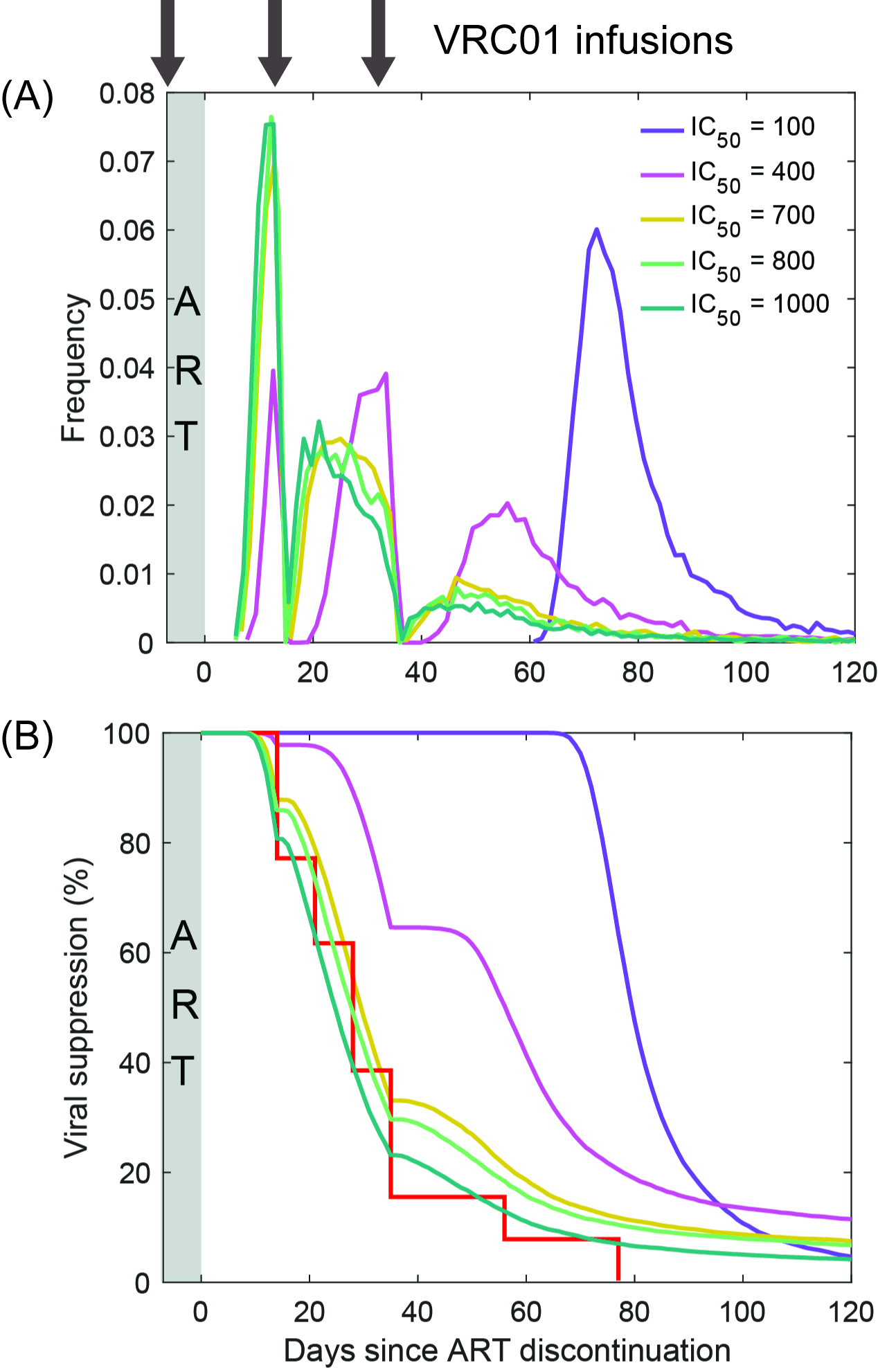

Supplement: S4 Fig — Model predictions similar to those in (A) Fig 4C and (B) Fig 4D with different values of the IC50 (μg/mL) indicated. The red line in (B) is data from the A5340 trial. (TIF) [file pcbi.1008434.s007.tif]

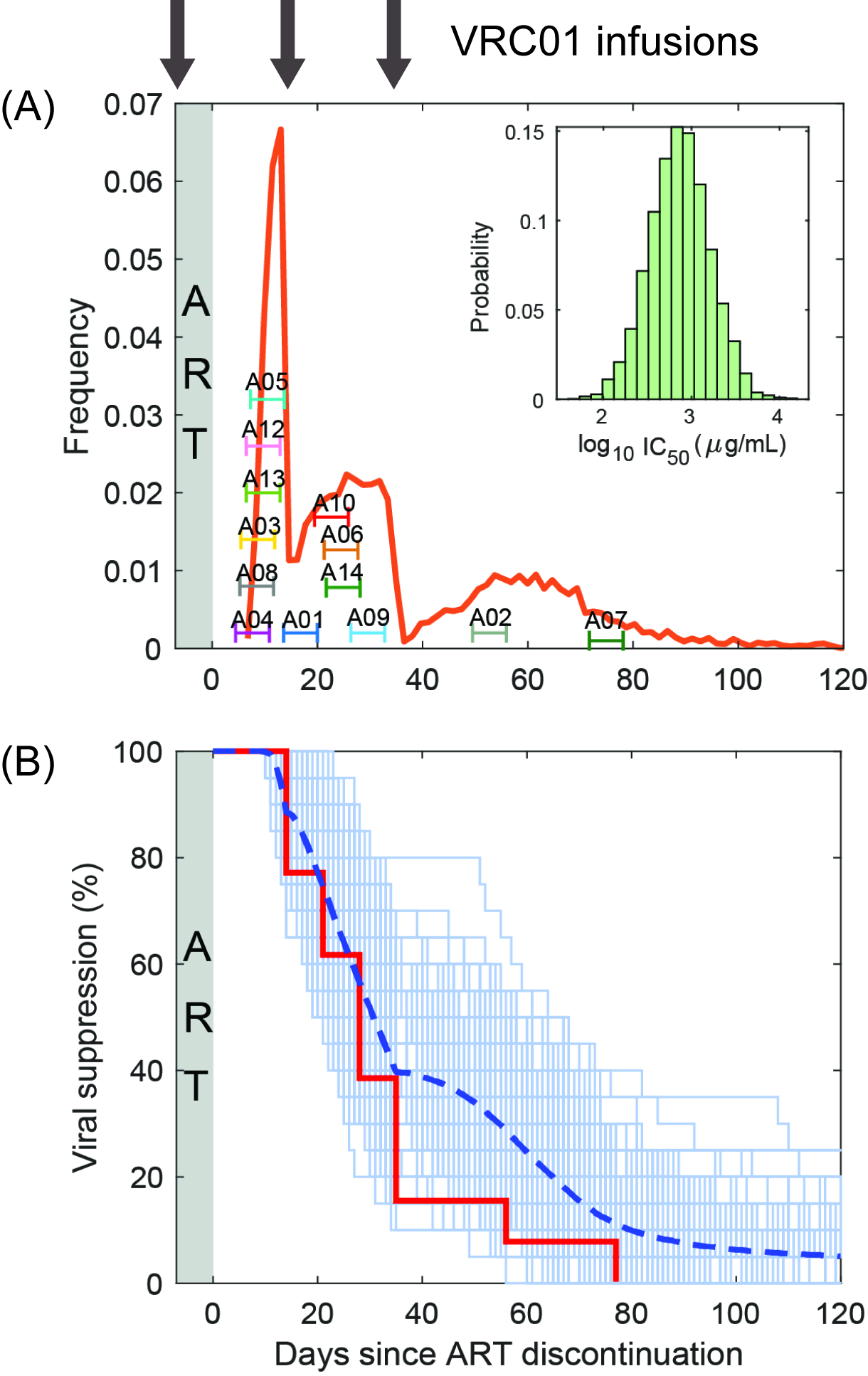

Supplement: S5 Fig — Model predictions similar to those in (A) Fig 4C and (B) Fig 4D but with the IC50 (instead of p) in each virtual patient drawn from a log-normal distribution (Log10IC50~N(2.86, 0.35)) (Inset). The red line in (B) is data from the A5340 trial. (TIF) [file pcbi.1008434.s008.tif]

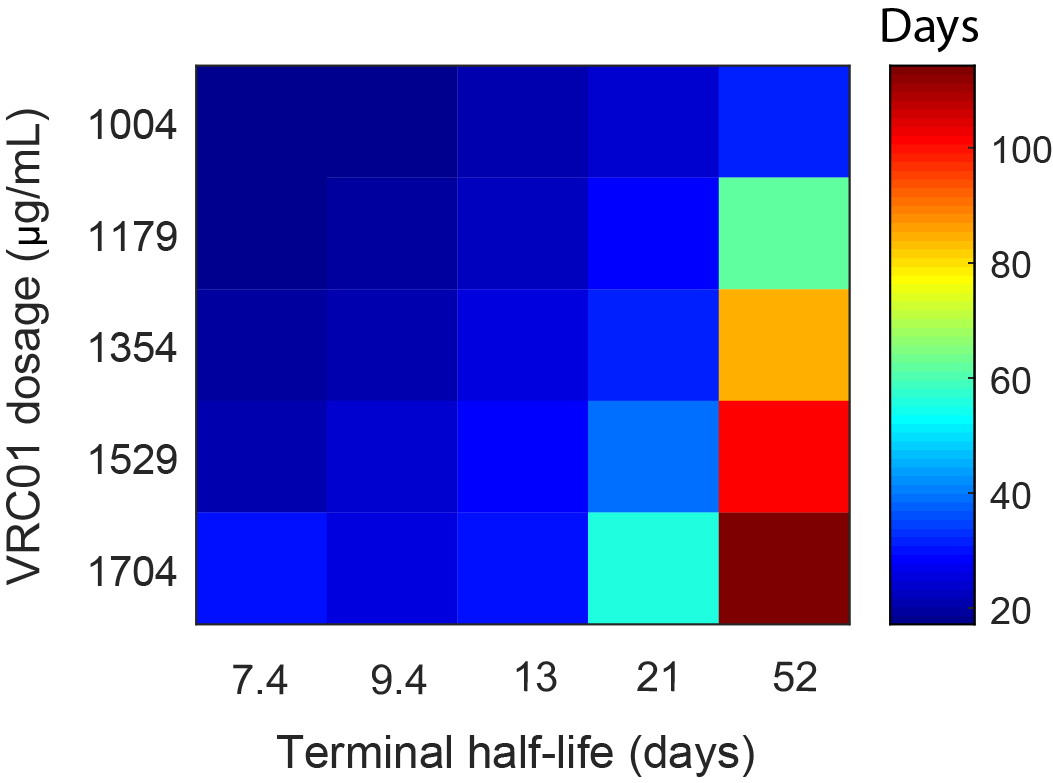

Supplement: S6 Fig — Heat map showing the median rebound time, corresponding to the detection limit of 20 copies/mL, for different VRC01 dosages and half-lives. For each parameter combination, a virtual population of 10000 individuals was employed following the dosing schedule in the A5340 schedule, as in Fig 4. Note that the modified bNAb VRC01LS has a >4-fold longer half-life than VRC01, and has been tested in healthy and uninfected adults for safety and pharmacokinetics [100]. The terminal half-life, η1, has been varied accordingly. The initial total antibody concentration (A1+A2) has been varied while keeping the ratioA1/A2 fixed, with the maximum (A1+A2) (in μg/mL) corresponding to the 40 mg/kg dosage of VRC01 (fitted from data [26]), which appears to be the maximum dosage of VRC01 reported. The other parameters are the same as in Fig 4. (TIF) [file pcbi.1008434.s009.tif]
